# Reactive Chemistry at the Unrestricted Coupled Cluster Level: High-Throughput Calculations for Training Machine Learning Potentials

**DOI:** 10.1021/acs.jctc.6c00247

**Published:** 2026-07-14

**Authors:** Alice E. A. Allen, Rui Li, Sakib Matin, Xing Zhang, Benjamin Nebgen, Nicholas Lubbers, Justin S. Smith, Richard Messerly, Sergei Tretiak, Garnet Kin-Lic Chan, Kipton Barros

**Affiliations:** † Center for Nonlinear Studies, 5112 Los Alamos National Laboratory, Los Alamos, New Mexico 87545, United States; ‡ Theoretical Division, Los Alamos National Laboratory, Los Alamos, New Mexico 87545, United States; § 28308 Max Planck Institute for Polymer Research, Ackermannweg 10, 55128 Mainz, Germany; ∥ Division of Chemistry and Chemical Engineering, California Institute of Technology, Pasadena, California 91125, United States; ⊥ Computer, Computational, and Statistical Sciences Division, Los Alamos National Laboratory, Los Alamos, New Mexico 87545, United States; # 196328 Nvidia CorpOration, 2788 San Tomas Expressway, Santa Clara, California 95051, United States; ¶ National Center for Computational Sciences Division, 6146 Oak Ridge National Laboratory, Oak Ridge, Tennessee 37830, United States; ∇ Center for Integrated Nanotechnologies, Los Alamos National Laboratory, Los Alamos, New Mexico 87545, United States

## Abstract

Modeling chemical reactions accurately at the atomistic level requires high-level electronic structure theory due to the presence of unpaired electrons and the need to properly describe the energetics of bond breaking and bond formation. Commonly used approaches such as density functional theory (DFT) frequently fail for this task due to deficiencies that are well recognized. However, for high-fidelity approaches, creating large data sets of energies and forces for reactive processes to train machine learning interatomic potentials (MLIPs) or force fields is daunting. For example, the use of the unrestricted coupled cluster level of theory has previously been seen as unfeasible due to high computational costs, the lack of analytical gradients in many computational codes, and additional challenges such as constructing suitable basis set corrections for forces. In this work, we develop new methods and workflows to overcome the challenges inherent to automating unrestricted coupled cluster calculations. Using these advancements, we create a data set of gas-phase reactions containing energies and forces for 3119 different organic molecules configurations calculated at the gold-standard level of unrestricted CCSD­(T) (coupled cluster singles doubles and perturbative triples). With this data set, we provide an analysis of the differences between the density functional and unrestricted CCSD­(T) descriptions. We develop a transferable MLIP for gas-phase reactions, trained on unrestricted CCSD­(T) data, and demonstrate the advantages of transitioning away from DFT data. Transitioning from training to DFT to training to UCCSD­(T) data sets yields an improvement of more than 0.1 eV/Å in force accuracy and over 0.1 eV in activation energy reproduction.

## Graphical abstract

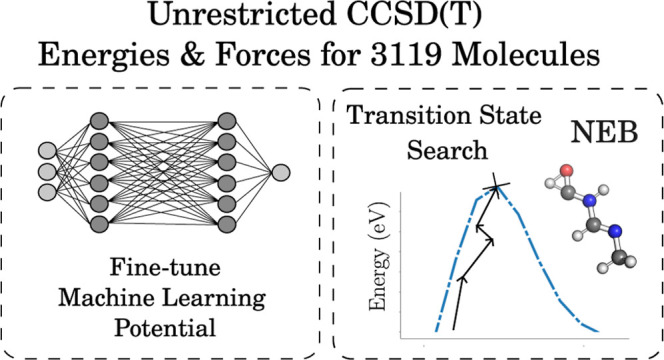

## 1 Introduction

Atomistic simulations of molecular systems underpin the understanding of a wide variety of biological and chemical processes.^1–5^ Simulating chemical reactions is consistently more challenging than handling nonreactive systems due to multiple factors. Reactive systems often involve modeling configurations with unrestricted formalisms to describe bond breaking and formation processes. Additionally, reactions typically feature highly diverse conformations across the potential energy surface (PES), with pathways and transition state (TS) barriers often unknown beforehand. Conquering these challenges is important, as precise knowledge of the TS energetics is necessary for understanding the probability and efficiency of reactions. Common quantum mechanical (QM) approximations can fail to describe the reaction paths sufficiently accurately, with significantly more expensive techniques beyond density functional theory (DFT) often required.^6–13^ Alternatively, machine learning interatomic potentials (MLIPs) can provide predictions of QM properties (such as energies and forces) that are accurate and orders of magnitude faster than high-level QM techniques.^4,14–19^ However, MLIPs must be derived from large data sets of QM calculations and therefore the issues associated with using QM calculations for reactive systems are still present when creating reactive MLIPs. In this work, we develop an automated workflow to create an organic molecule gas-phase data set using high-level QM data from the unrestricted coupled cluster singles doubles and perturbative triples (CCSD­(T)) approximation. We perform analysis on this data set to gain insight into the differences between unrestricted CCSD­(T) (UCCSD­(T)) and DFT approximations across a range of chemical reaction space expressed in the training data. Finally, by training an MLIP to the UCCSD­(T) data set and subsequent evaluation of its performance, we demonstrate that transferable MLIPs can be created for reactive chemistry that reproduce high accuracy training data.

Coupled cluster theory is commonly used for accurate ground-state calculations on small molecules at equilibrium geometries where it is referred to as the gold-standard of quantum chemistry.^20^ Furthermore, the need for high-fidelity coupled cluster data for studying reaction dynamics is well-known within the field.^21–23^ In its unrestricted formulation, it also provides reasonably accurate results for typical chemical bond breaking, e.g., those involving single bonds.^20,24^ Since the goal of MLIPs is often to replicate a system’s ground-state energy, unrestricted calculations offer a practical route to accurate data for reactive systems. The discrepancies between the predictions of DFT and CCSD­(T) for reactive systems have been investigated in a number of previous studies.^6–13,25^ These studies looked at a small subset of reaction space, consisting of a few chosen reactions, and investigated differences in the estimated activation energy and transition states between DFT functionals and CCSD­(T).^8,13,26^ In this work, we are interested in exploring the difference between DFT (for three commonly used functionals) and UCCSD­(T) for a broader range of reactive chemistry across hundreds of different reactions, and investigating the consequences of the level of theory used for MLIP development. The resulting UCCSD­(T) data set contains the energies and forces for thousands of molecules and forms the starting point in this work for creating a transferable and high-accuracy machine learning model capable of modeling a wide range of reaction space.

Running coupled-cluster calculations at scale requires new considerations beyond those typical in studies of a limited number of molecules. For example, UCCSD­(T) calculations should use an appropriate Hartree–Fock reference (i.e., one that breaks spin symmetry) if such an unrestricted determinant exists. To enable high-throughput data collection, we automate the construction and selection of the unrestricted Hartree–Fock reference using stability analysis.^27^ Because CCSD­(T) requires large basis sets for chemical accuracy, we also employ a basis set correction for both the energy and the forces.^28^ We provide evidence of the effectiveness of the basis set correction for our data set. Finally, the UCCSD­(T) data can be unreliable at points close to the boundary of the Hartree–Fock symmetry-breaking point. Thus, we also developed an automatic filtering protocol to remove structures where the UCCSD­(T) result is suspected to be inaccurate.

The choice of sampling method for exploring chemical space plays a crucial role in constructing a high-quality QM data set for MLIPs. Here, we focus on building a data set for molecules containing C, H, N, O atoms and use active learning to select relevant structures in the chemical reaction space.^29^ We start from a subset of reactants, products, and transition states of molecules in existing data sets in the literature^30^ and subsequently employ several automated structure sampling techniques to generate new structures.^31–33^ By using an ensemble of exploratory MLIP, we detect high uncertainty points among these structures to identify points in chemical space to add to the data set.^29^ Then by using transfer-learning from a MLIP trained to DFT energies and forces on these actively learned points, we build a final MLIP on high-quality UCCSD­(T) energies and forces on a smaller actively learned set of structures.^3^ Our final UCCSD­(T) data set consists of the energies and forces of over three thousand sampled configurations for molecules with up to 16 atoms.

To put our MLIP work in context, MLIPs have previously been fit to large, diverse data sets of organic molecules to create transferable potentials capable of modeling a wide range of chemical and biological systems.^4,29,34,35^ Furthermore, this has been extended to reactive systems in ref 36 where high-temperature molecular dynamics was performed to sample reaction space in a “nanoreactor”. Additionally, in ref 37 gas-phase reactive data sets were created and fit to model reactive systems. However, these potentials have relied on DFT training data which fundamentally limits the accuracy of the potentials. In ref 38, a foundation model fit to the Materials Project data set was created,^39^ which successfully recreated hydrogen combustion reactions, ammonia and borane thermal decomposition, and additional reactive processes. However, given that the Materials Project data set is also generated at the DFT level, the QM level of theory is a fundamental limitation in the accuracy obtainable with this model.^38^

For nontransferable MLIPs, a number of studies have employed CCSD­(T) data, or data from even more expensive quantum chemistry methods.^21,40–42^ For example, permutationally invariant polynomials have been fit to small-molecule potential energy surfaces at the CCSD­(T) level, with specialized software developed to facilitate this process.^21,23,43,44^ Permutationally invariant polynomial models have been used to study a variety of phenomena from mode-specific reaction dynamics to roaming dynamics.^21,22^ Delta learning and transfer learning approaches targeting CCSD­(T) accuracy have also been explored for individual systems. For instance, ref 45 used transfer learning to develop a water MLIP at the CCSD­(T) level of theory, while ref 46 employed a delta learning approach to achieve CCSD­(T) level accuracy for liquid water. However, these models are designed for a single or limited number of applications and lack transferability, meaning that each new application requires the generation of additional CCSD­(T) data and the training of a new MLIP.^21,40–42^ Ideally, the community would have a transferable MLIP capable of reaching CCSD­(T) accuracy without the need for further data generation.

In this work, our intention is to provide a large organic molecule data set of UCCSD­(T) energy and forces for reactive processes and create a transferable MLIP that can be used on many different organic reactions. This is a significant step toward creating transferable MLIP that can describe even broader classes of reactions, for example, both in the gas phase and condensed phase, at the coupled cluster level of accuracy.

## 2 Methods

### 2.1 MLIP Framework with HIP-NN

MLIPs predict the energy and forces of a molecule from atomic coordinates.^4,29,34,35^ MLIPs are fit to QM calculations and can offer highly accurate and very fast models of interactions between atoms and molecules. In this work, we used the model HIP-NN in two architectures.^47,48^ The first was used for exploring reaction space using active learning. For this, we used HIP-NN-TS, a messaging-passing neural network with tensor sensitivity.^47,48^ The hyperparameters are set to the same values used in ref 47 used for training the HIP-NN-TS potential to the ANI-1x data set. To calculate the uncertainty, an ensemble of four HIP-NN-TS potentials was trained, and the standard deviation of the energy (per atom) was used as the uncertainty for the active learning process.

The second type of MLIP was the one trained to the final data sets of DFT and UCCSD­(T) energies and forces. For this, the newest version of the HIP-NN class of models was employed. This model, HIP-HOP-NN, is introduced in ref 49 and incorporates many-body tensor information directly into the message-passing components of the model. An ensemble of 8 HIP-HOP-NN models was used to improve the accuracy of the model when predicting energies and forces. We used transfer learning^3,50^ by first fitting the MLIP to the (larger) DFT data set, before fine-tuning to the smaller UCCSD­(T) data set.

By training on UCCSD­(T) data, the MLIP learns a single PES at UCCSD­(T). No explicit spin states are present in the model, instead, we fit to the lowest energy state for a given atomic configuration.

### 2.2 Existing Data Sets Used for Initialization

QM data sets of molecular energies and forces are necessary for training MLIPs. For our training data, we started from subsets of structures in existing data sets from the literature, which we then augmented with new structures that sample reaction space generated by the active learning sampling protocol. In the following section, we describe the existing data sets used to initialize the sampling process. Note that although we used structural data from these data sets, we always recomputed the energies and forces using the QM methods of this work.

#### 2.2.1 Reaction Paths from Ref 30

In ref 30, ≈12,000 reaction pathways were discovered using the single-ended growing string (SEGS) method to suggest products of a given reactant.^33^ The molecules chosen contained C, H, N, O elements. The minimum energy path and TS were subsequently found, and the reactants, products, and TS located using the ωB97X-D3/def2-TZVP level of theory (all in the singlet state). The use of restricted DFT calculations for this data set has subsequently been questioned in ref 51 due to the presence of strong electron correlations.

#### 2.2.2 Transition-1x

In the Transition-1x^37^ data set, the data set of ref 30 was used to initialize points along the minimum energy paths for ≈10,000 reactions. Again, the molecules chosen contained C, H, N, O elements. The Transition-1x data set was constructed from the structures encountered as NEB attempted to find a converged reaction path from the ref 30 reaction paths.^32^ The level of theory used was DFT with ωB97X/6–31G­(d), and the data set consisted of 9.6 million configurations.

#### 2.2.3 QM9

The QM9 data set was first introduced in ref 52. The QM9 data set contains 133,885 organic molecules with up to nine heavy atoms and contains the elements C, H, O, N, F. The organic molecules contained are equilibrium configurations calculated at the B3LYP/6–31G­(2df,p) level of theory.^53–56^

### 2.3 QM Methods

Additional quantum-mechanical calculations were then performed at both DFT and coupled cluster levels of theory. We now describe the QM methods used to generate the energy and force data. To facilitate the extension of these data sets, the Python code to carry out the calculations used in this work is available as part of the active learning framework (ALF) code at https://github.com/lanl/ALF.

#### 2.3.1 DFT Calculations

The DFT calculations were performed on structures produced using the existing data sets above as well as new structures produced by active learning. The calculations used the ORCA package at the ωB97X/6–31G­(d) level of theory.^57^ All DFT calculations were carried out in the singlet state using restricted Kohn–Sham theory, as commonly performed for reactive data sets constructed for MLIPs.^30,37^ The B3LYP and PBE0 calculations were also evaluated with the 6–31G­(d) basis set using the ORCA package.

#### 2.3.2 UCCSD­(T) Calculations

The UCCSD­(T) data were computed with PySCF^58,59^ with the cc-pVDZ (DZ) basis set alongside a basis set correction obtained from calculations performed at the UMP2/QZ, UMP2/TZ, UCCSD/TZ, and UCCSD/DZ levels. These data were combined to estimate the UCCSD­(T) QZ energies and forces. The basis set corrected UCCSD­(T) is referred to as UCCSD­(T)/QZ* throughout this work. We used a composite scheme with the following form for the energies and forces

1 $$\begin{array}{l} E_{UCCSD\left(T\right)/QZ*}=E_{UCCSD\left(T\right)/DZ}+\\ \left(E_{UCCSD/TZ}-E_{UCCSD/DZ}\right)+\\ \left(E_{UMP2/QZ}-E_{UMP2/TZ}\right) \end{array}$$

2 $$\begin{array}{l} F_{UCCSD\left(T\right)/QZ*}=F_{UCCSD\left(T\right)/DZ}+\\ \left(F_{UCCSD/TZ}-F_{UCCSD/DZ}\right)+\\ \left(F_{UMP2/QZ}-F_{UMP2/TZ}\right) \end{array}$$

The error of the correction compared to the higher level UCCSD­(T)/QZ forces is 0.073 eV/Å per force component which is smaller than the model errors in this work. Other aspects of this correction are analyzed in detail in Section S1.

**Figure 1:**
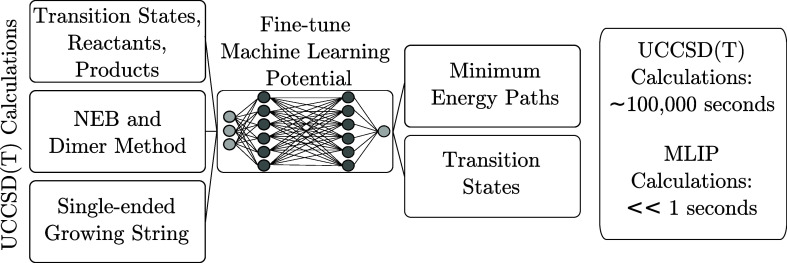
Unrestricted CCSD­(T) calculations sampled with a variety of methods are used to fine-tune a MLIP pretrained to DFT data. The subsequent MLIP can then predict minimum energy paths and transition states of reactions beyond the training data.

The effect of the basis set correction used is visualized in Figure 2 with a comparison of UCCSD­(T)/QZ* and UCCSD­(T)/DZ forces. UCCSD­(T)/DZ forces show a large deviation from the UCCSD­(T)/QZ* with systematically larger forces for the DZ basis than the QZ* basis. The correlation of the components used in the basis set correction is shown in Figures S1 and S2.

**Figure 2:**
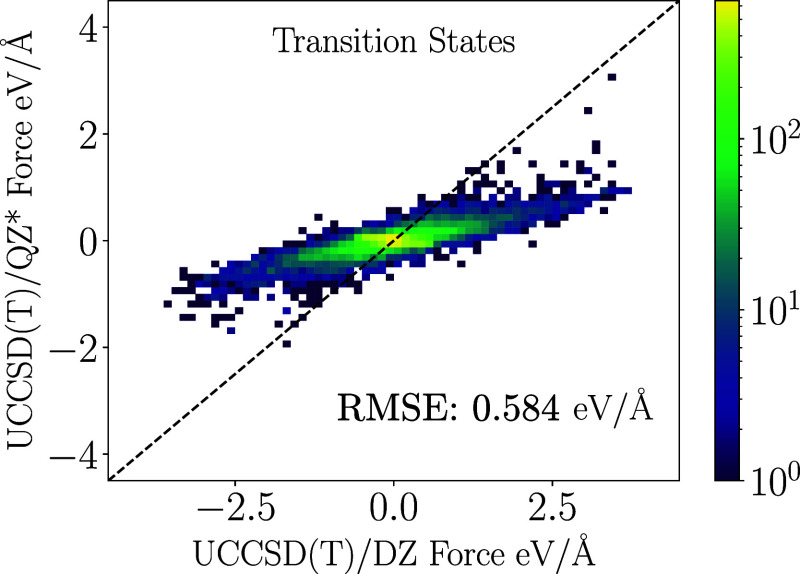
To demonstrate the importance of basis set corrections, the forces for UCCSD­(T)/DZ compared to the UCCSD­(T) forces with a basis set correction up to the QZ level is shown. A systematic error in DZ forces can be seen, demonstrating the importance of basis set corrections and the insufficiency of using UCCSD­(T)/DZ for force calculations or geometry optimization.

To perform the UCCSD­(T) calculations, we should start from an appropriate Hartree–Fock reference. Near the equilibrium geometry, for an even number of electrons, the restricted Hartree–Fock reference with a singlet ground state, where opposite spin electrons occupy the same spatial orbitals, is commonly the lowest energy solution. However, at some geometries (typically stretched ones), the restricted Hartree–Fock solutions can develop an instability toward an unrestricted solution, where opposite spin electrons occupy different spatial orbitals. We used stability analysis and following of the Hartree–Fock (HF) wave function, as presented in ref 27, to detect instabilities in the HF solution and find stable unrestricted HF (UHF) solutions. Finding the stable ground-state solution for the HF calculation ensures that the electronic structure remains as single reference as possible (by being in the ground state) even at stretched geometries, which is a prerequisite for the accuracy of subsequent UCCSD (T) calculations.^60^

However, there are some complications when using unrestricted coupled cluster energies. One issue is that the unrestricted wave function is no longer guaranteed to be an eigenvalue of the *S* ^2^ operator.^61^ In practice, this spin contamination is not necessarily a concern for the quality of the predicted energies. However, a more problematic issue is that at the boundary between geometries with unrestricted Hartree–Fock and restricted Hartree–Fock solutions (the Coulson-Fischer points^62^), the energy is continuous but not differentiable, thus the force is undefined. This means that forces are inaccurate near this boundary of the Hartree–Fock instability. This issue is exacerbated when using the basis set correction as the boundary can manifest at slightly different geometries depending on the basis set, making calculations with different basis sets inconsistent.

To remove these complications, we use a filtering procedure to detect when the geometry is too close to the Coulson-Fischer point and to remove such structures. A detailed description of the procedure is in Section S1. To demonstrate the type of configuration removed in this process, an example structure removed from the data set is shown by the TS in ref 3 along with the reactants and products. This reaction is an example of the unusual reactions created with automated approaches for reaction paths. Although the central structure in Figure 3 is suggested to be a TS in the DFT simulations, for UCCSD/TZ much higher forces are present, up to 11.12 eV/Å. Similarly, the MP2 calculation exhibits large forces. For the UHF references using the DZ/TZ/QZ basis sets, ⟨*S* ^2^⟩ = 0.51/0.22/0.13 demonstrating that the ⟨*S* ^2^⟩ values are basis set dependent. The TS structure occurs near the point where the 5-membered ring breaks, which likely produces a diradical. The lowest eigenvalue of the orbital Hessian of this structure is <10^–3^ Ha, indicating closeness to the Hartree–Fock instability, which is leading to the inaccurate forces.

**Figure 3:**
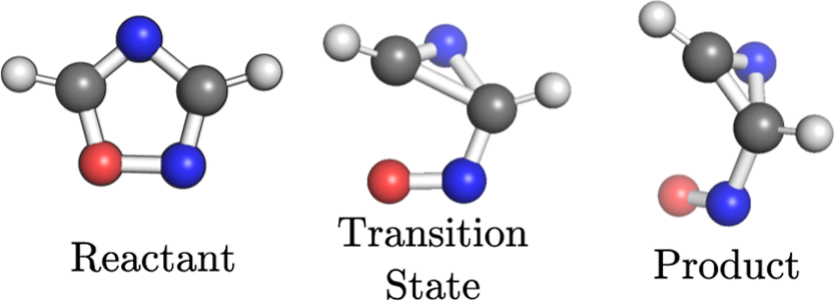
Reactants, products, and transition states for an example of an excluded TS structure in the data set. The forces for the MP2 and UCCSD/TZ calculations are very high for this nominal TS (up to 11.12 eV/Å for one force component).

### 2.4 Sampling Methods for Data Set Construction

When generating molecular data sets to train MLIPs, there are two considerations: the quantum-mechanical approximation used and the sampling method employed. The sampling method is a technique used to generate molecular structures. To construct our training data set, we used different sampling methods, initialized from structures in the existing data sets above, to efficiently explore reactive space (see Figure 1). We first created a larger data set with energies and forces computed at the DFT level and then used transfer learning to generate UCCSD­(T) data.

We have provided the sampling techniques for active learning with the dimer method and NEB within the MLIP ALF available at https://github.com/lanl/ALF.^36,63^ The SEGS method is freely available in ref 33 with code additions to automate sampling presented in ref 30. In this work, we used the existing SEGS software with minor additions to enable MLIPs to be incorporated.

#### 2.4.1 AL for the DFT Data Set

Active learning uses the uncertainty of an ML model to select molecular structures to be added to the training data. This reduces the computational cost of data set generation. The active learning strategy for the DFT data set is as follows.

To carry out the sampling, an exploratory MLIP was first trained to DFT data computed for 10% of the Transition-1x structures. Then, from the transition states in ref 30, the dimer method was used with the exploratory MLIP to find the MLIP-optimized TS.^31^ If the proposed TS had an uncertainty greater than a given threshold, the structure was calculated with DFT and added to the new data set. The number of steps allowed for the dimer method was limited to maintain realistic structures that did not differ significantly from the starting point.

Further, using the reactants and products from ref 30, approximate minimum energy paths were calculated using the NEB method using the exploratory MLIP.^32^ If the structures proposed in the final set of structures had an uncertainty above a set threshold, they were calculated with DFT and added to a new data set. The NEB was not necessarily converged at this final stage, and this means that both the true minimum energy path and regions around it were sampled.

Finally, the SEGS method together with the exploratory MLIP was also used to sample reactive pathways^33^ beyond those in ref 30. This method relies on only a reactant and driving coordinates that state the new bonds to be formed and broken. The existing QM9 data set of equilibrium molecules from ref 52 was used to suggest reactants, and the driving coordinates were randomly chosen using the code provided in ref 30. The QM9 reactants and driving coordinates were then fed into SEGS software. Again, QM calculations were performed if the uncertainty was above a set threshold.

The total size of the DFT data set sampled in this way consisted of 270,720 structures, with a breakdown of structures from each of the sampling techniques given in Section S4. The final HIP–HOP DFT was trained to this data set in addition to 10% of Transition-1x.

#### 2.4.2 AL for the UCCSD­(T) Data Set

The active learning strategy for the UCCSD­(T) data set is now described. In the present work, the molecules from ref 30 were used to build an initial data set of reactants, products, and transition states at the UCCSD­(T) level for 950 molecules. Transfer learning was used to retrain the exploratory DFT MLIP to the data set of transition states, reactants, and products to create an exploratory coupled cluster MLIP.

With this exploratory coupled cluster MLIP, sampling with the dimer method, NEB sampling, and the SEGS method was again performed. This provided additional samples to extend the UCCSD­(T) data set to points off the minimum energy path.

An additional set of molecules was also added with bonds stretched or compressed to large and small values by randomly sampling the bond lengths. These molecules were added to improve the bond dissociation curves and repulsive regions at small distances. The molecules chosen for this were primarily very small molecules with fewer than 7 atoms.

From the active learning process, 167 configurations were found from the dimer sampling method, and 49 configurations were found with the SEGS method. Additionally, 1953 small molecule structures (<9 atoms) were added with bond lengths stretched, as described above. The final UCCSD­(T) data set consisted of 3119 configurations. A summary of the data set created is given in Section S4. The final HIP–HOP UCCSD­(T) MLIP was trained to this data set by transfer learning from the DFT HIP–HOP potential.

## 3 Results

The creation of a UCCSD­(T) data set offers the unique opportunity to both analyze the differences between QM levels of theory and demonstrate the improved performance of MLIPs fit to UCCSD­(T) data. We begin by comparing UCCSD­(T) data with DFT data before examining an MLIP fit to the UCCSD­(T) data.

### 3.1 Analyzing the UCCSD­(T) Data Set

The UCCSD­(T)/QZ* data set enables an analysis of the differences between UCCSD­(T) and DFT quality data across a broad range of chemical reactions. We first focus on the reactants, products, and transition states before considering the other sampled geometries. There are 950 structures that are reactants, products, and transition states.

#### 3.1.1 Reactants, Products, and Transition States

The forces for three DFT functionals, ωB97*x*/6–31G­(d), PBE0, and B3LYP, compared to those from UCCSD­(T), are shown in Figure 4. Note that these are all evaluated at the structures taken from existing data sets, which were optimized with a different level of theory (ωB97X-D3/def2-TZVP); thus, we do not expect the forces to vanish. Nevertheless, all the forces are highly concentrated around zero. Different levels of theory are often used for geometry optimization and the final energy and force computation during data set construction, and this analysis supports that protocol.

**Figure 4:**
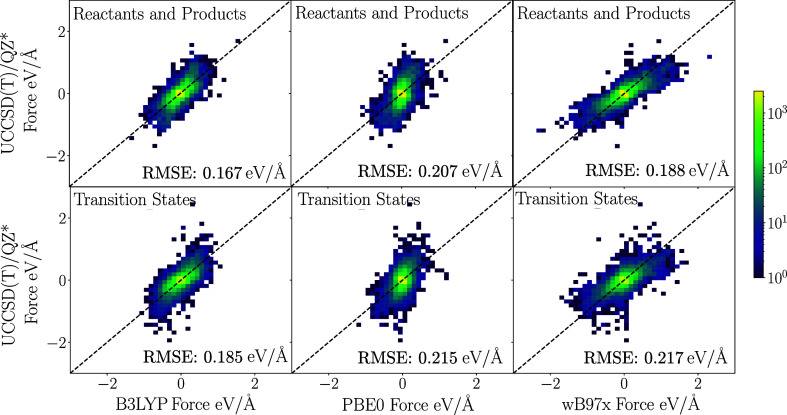
A comparison of forces for DFT functionals calculated in the singlet state and UCCSD­(T)/QZ* for the transition states and products and reactants. Consistently higher RMSE is seen in the transition states over the reactants and products.

However, nonvanishing forces do occur, and the deviations between these forces for the various DFT functionals and UCCSD­(T) have some spread. Figure S6 demonstrates this with the distribution of force errors shown for each DFT functional.

The activation energies of the reactions for three DFT functionals are compared to UCCSD­(T)/QZ* in Figure 5 (note, the activation energies are calculated using the TS from original data set optimized at DFT/ωB97X-D3/def2-TZVP level). We see some systematic trends: B3LYP overestimate the barrier height (versus UCCSD­(T)/QZ*), PBE0 has the best correlation but still has a systematic overestimation, and ωB97X/6–31G­(d) underpredicts the barrier height, as reflected by the equation of the line of best fit through the data points. The difference in activation energy between CCSD­(T) and DFT has been highlighted in ref 64 and will cause significant differences in calculated properties such as reaction rates.

**Figure 5:**
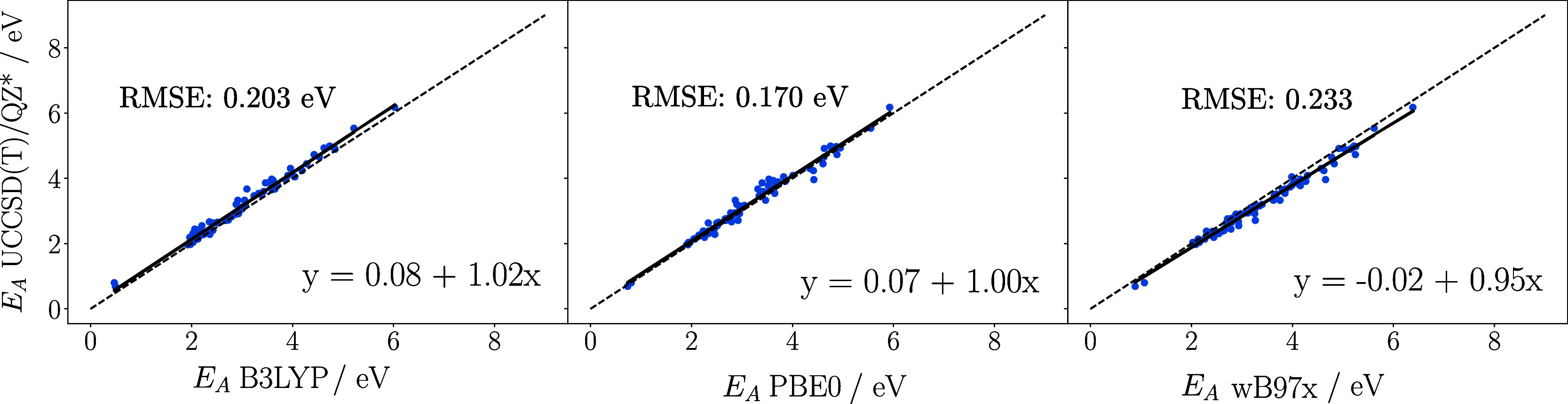
Activation energy for DFT functionals in comparison to UCCSD­(T)/QZ* activation energies. The structures used for the TS, reactants, and products are optimized at the ωB97X-D3/def2-TZVP level and calculated in the singlet state. The dashed black line shows *y* = *x*, and the solid black line gives the line of best fit through the points with the corresponding equation shown in each figure.

#### 3.1.2 Data Set Constructed along the Reaction Path

Next, we analyzed the data for structures generated by sampling along the reaction path. There are 216 of these structures in the data set. A comparison between the DFT and UCCSD­(T) forces is shown in Figure S12, and the distribution of UCCSD­(T)/QZ* forces is shown in Figure S11. For the transition states, reactants, and products, the distribution is centered around zero, as expected. For the structures sampled with the NEB and dimer methods and the SEGS method, a much larger range of forces are sampled, and we can conclude that the diversity of chemical space is increased by the addition of these structures.

For structures sampled by the NEB dimer method, the force component RMSE is 0.470/0.466/0.498 eV/Å for the B3LYP/PBE0/ωB97X functional. For the SEGS method, the force component RMSE is 0.746/0.734/0.812 eV/Å for the B3LYP/PBE0/ωB97X functional. In addition, the DFT data have different slopes compared to the reference UCCSD­(T) data: the fitted slopes are between 0.85 and 0.92 and larger deviations between DFT and UCCSD­(T) forces occur as the magnitude of the force component increases. Overall, the force error for the DFT functionals for structures far from equilibrium is greater than for the equilibrium structures. This highlights the importance of high-quality data for off-equilibrium calculations when studying reaction paths, mechanisms, and performing free-energy calculations. It is also worth noting that the deviations between DFT and UCCSD­(T) forces reported here are partly a consequence of this basis set choice, and better agreement may be expected with a larger basis set.

### 3.2 Fitting Transferable MLIPs to UCCSD­(T) Data

We now report the performance of the MLIP trained on the DFT and UCCSD­(T) data sets using the latest version of the HIP-NN model, HIP-HOP-NN.^49^ The computational cost of a force evaluation for a small molecule (less than 20 atoms) for the HIP-HOP-NN model is approximately 15 ms on an A100 GPU.

Figure 6 demonstrates the level of accuracy that is achievable with MLIPs. In Figure 6a, HIP-HOP-NN is trained to UCCSD­(T) data and for this model, the force recreation for the transition states, products, and reactants improves compared to the HIP-HOP-NN model trained to DFT data when evaluated against the UCCSD­(T) reference. In contrast, as depicted in Figure 6b, the HIP-HOP-NN model trained on ωB97x DFT data has nearly double the error on the UCCSD­(T) test set. For a test set representing the entire training data set, the baseline MLIP trained on DFT data achieves a force RMSE of 0.460 eV/Å for ωB97x and 0.500 eV/Å for UCCSD­(T). To put the reported numbers into context, the force errors report for subsets of SPICE with MACE-OFF range from 0.1 to 0.7 eV/Å.

**Figure 6:**
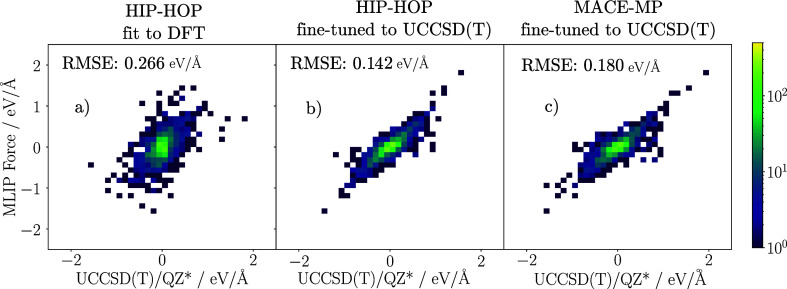
A comparison of forces calculated with the MLIP HIP-HOP-NN and UCCSD­(T)/QZ* for testing data sets containing the transition states and products and reactants (TS, P, R). Part (a) HIP-HOP-NN trained to DFT data only, (b) HIP-HOP-NN model trained to UCCSD­(T), and (c) is the foundation model MACE-MP fine-tuned to UCCSD­(T) data. When a MLIP is trained to the UCCSD­(T) data set, comparable (or better) error than the DFT functionals previously shown is achievable.

The energy error for the HIP-HOP-NN model also provides a measure of the accuracy of the potentials. The test set energy errors for the data set containing the TS, P, R, and AL structures is 0.0134 eV/atom for HIP-HOP-NN trained to UCCSD­(T). The error in the activation energy recreation also provides an informative measure of the utility of the potential; see Figure 7. The reaction paths chosen for the activation energies are not included in the UCCSD­(T) training data used to train the model. The MLIP is able to recreate the activation energies of the reactions with an RMSE of 0.252 eV. This exceeds the performance of the more-expensive QM DFT calculation shown. This highlights that for both recreation of forces and activation energies, training to UCCSD­(T) data can significantly improve the performance.

**Figure 7:**
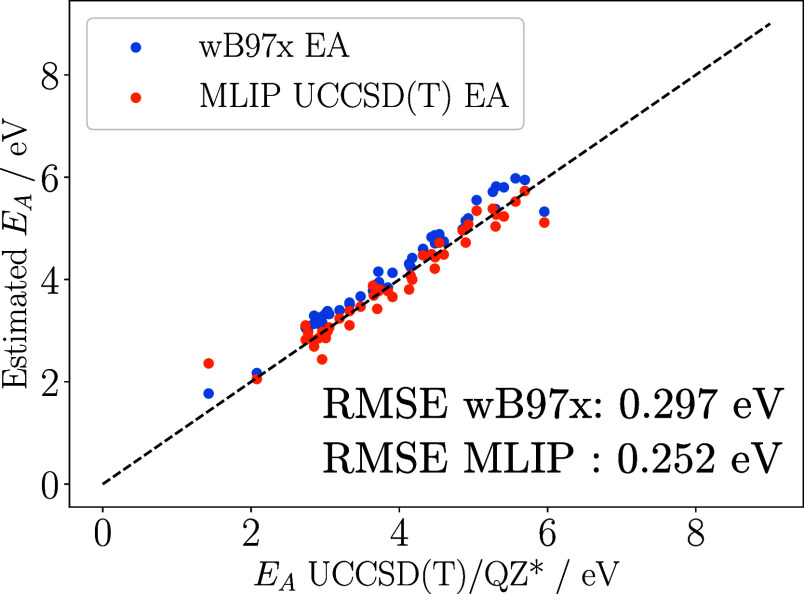
Activation energies for the HIP-HOP-NN model trained to UCCSD­(T) data compared to the ωB97x DFT functional. Reactions are taken from ref 30 and are not included in the UCCSD­(T) training data set. The HIP-HOP-NN model trained to ωB97x has an RMSE of 0.377 eV for the UCCSD­(T) activation energies and 0.179 eV for the ωB97x activation energies.

Foundation models have been developed by a number of groups to provide pretrained MLIPs that are transferable across multiple systems and can be fine-tuned to specific systems of interest. In preliminary work, we evaluated the performance of the MACE-OFF model in reproducing minimum energy pathways (MEP) and bond dissociation energies (BDEs). However, because MACE-OFF is not trained on reactive systems or transition states, its accuracy was insufficient for use as a pretrained model for our purposes. Instead, the MACE-MP model was found to produce smooth, well-converged MEPs and BDEs, likely due to its training on the more diverse Materials Project data set of DFT calculations. The error of the MACE-MP foundation model without fine-tuning has a large RMSE in the forces of 0.770 eV/Å. We can fine-tune this model to the UCCSD­(T) data set (see Sec. S5 for training details) to illustrate the performance of a different training method and model. We find that the fine-tuned model achieves a similarly low force error to the HIP-HOP-NN model trained with transfer learning, of 0.180 eV/Å.

We further used the HIP-HOP-NN MLIP to explore the minimum energy reaction paths with the NEB and dimer methods previously discussed. For this, we chose reaction paths that did not have any UCCSD­(T) data in the training set. The dimer methods initiated from the DFT TS structures consistently converged, as did the NEB method over the reaction paths. Example transition states found with HIP-HOP-NN UCCSD­(T) model are given in Figure S14. The MLIP consistently finds TS nearby to those proposed by the DFT method used in the Transition-1x data set and of similar quality with respect to the true UCCSD­(T) TS. Furthermore, the UCCSD­(T) forces of the TS found with the MLIPs were calculated and are shown in Figure S15. The MLIP trained to the UCCSD­(T) outperforms the MLIP trained to the DFT data, but the TS found with DFT still outperforms the MLIPs. This could be a consequence of the limited UCCSD­(T) data.

As an additional test of extrapolation capabilities, the bond dissociation curves for hydrogen abstraction for 11 randomly chosen molecules from QM9 were calculated with MLIP. All of the molecules chosen had more than 16 atoms and were larger than the molecules included in the UCCSD­(T) training data. The curves and BDEs are shown in Figure 8. The UCCSD­(T) BDEs are also shown and compared with the MLIP values and singlet DFT values. Relatively smooth bond dissociation curves were generated with an average RMSE of 0.485 eV in the bond dissociation energy achieved. The large discrepancy between the singlet DFT and UCCSD­(T) is also clearly shown. Given that BDEs were not the focus of this work, the results are promising. Further fragmentation data will be added in future iterations of the data set to improve performance as the bond dissociation error currently seen remains large. Additionally, proton transfer in malonaldehyde and tropolone was investigated, with MEPs found using NEB and molecular dynamics simulations. While smooth paths and physically reasonable behavior were observed for both molecules, quantitative agreement with the CCSD­(T) data was not achieved.

**Figure 8:**
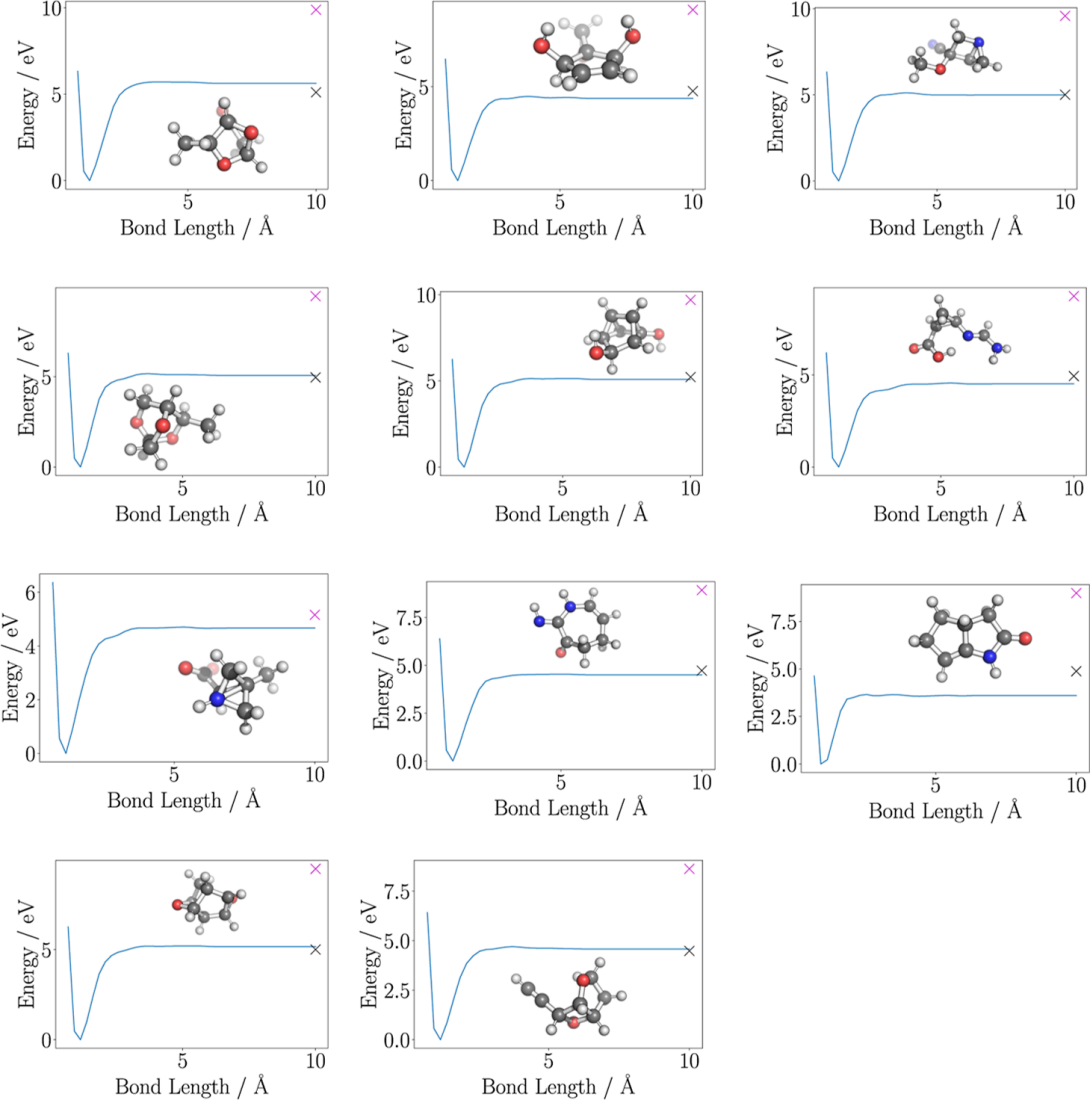
Hydrogen BDEs given by the MLIP for randomly chosen molecules from the QM9. The black cross shows the UCCSD­(T) bond dissociation energy relative to the equilibrium value. The magenta cross shows the DFT singlet bond dissociation energy. All of the molecules chosen are larger than the largest molecule used in the training data set and rigid scans are performed.

As a final demonstration of the MLIP, minimum energy paths and TS states for two isomerization reactions are given in Figure 9. The HCN to HNC isomerization in Figure 9a shows a smooth reaction path with NEB readily converging. The MLIP barrier height is above the UCCSD­(T) and DFT energies (0.467 eV greater), but the product energy is very close to that of the UCCSD­(T) energy (0.024 eV). This highlights the need for further energy calculations for transition states, including for small systems, in future work. The reaction path in Figure 9b demonstrates a cyclic isomerization reaction from ref 65. The molecules involved are larger than those included in the training data set. Again, the MLIP-calculated minimum energy path follows a similar shape to that of the DFT calculation, with some overestimation of the reaction barrier height. For the cyclic isomerization reaction from ref 65, the MLIP also recreates the reaction mechanism with the proton transfer occurring after the TS. Note that the NEB and dimer methods using an MLIP take minutes to perform for this system but are prohibitively expensive with UCCSD­(T)/QZ*.

**Figure 9:**
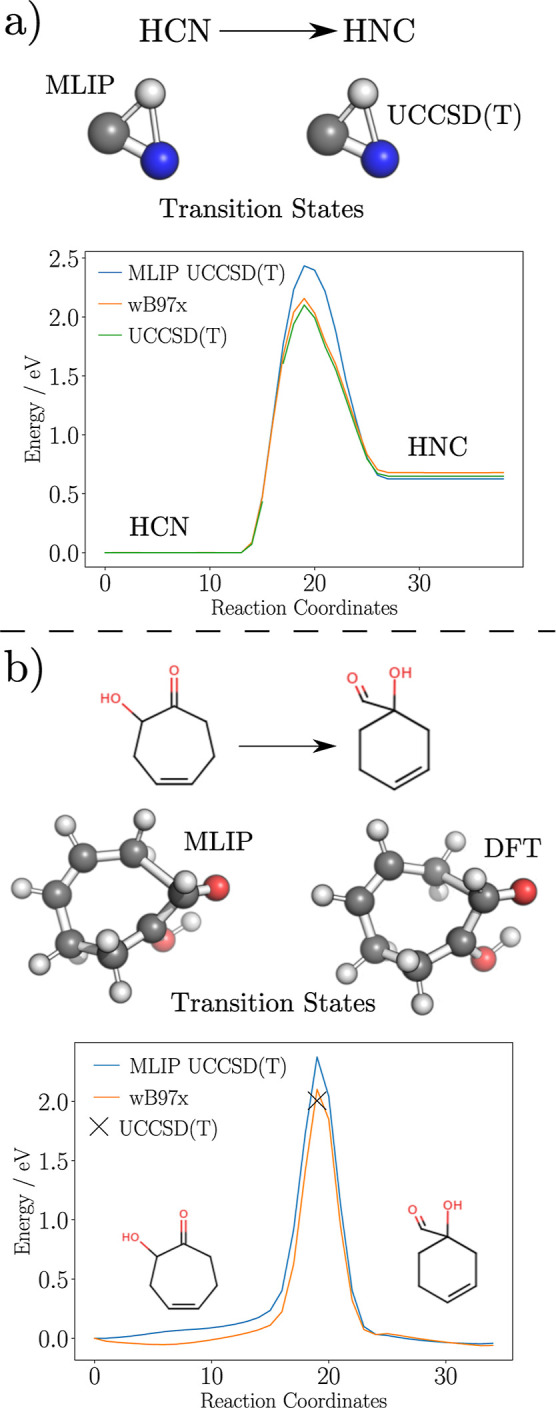
Transition states and change in energy along the reaction coordinate with the MLIP trained to UCCSD­(T) for (a) HCN isomerization and (b) cyclic isomerization reaction from ref 65. The TS is (a) the UCCSD­(T) TS for HCN isomerization and (b) a DFT TS for the cyclic isomerization reaction.

## 4 Conclusion

Modeling reactive processes with atomistic simulation poses an ongoing challenge for the computational chemistry community. While MLIPs provide a fast and accurate model, creating data sets of highly accurate QM calculations for training transferable MLIPs has remained an open challenge. We have demonstrated that analytical force and energy calculations at the UCCSD­(T) level can be performed at a large-scale and in an automated way in order to generate data sets for machine learning. Some practical adaptations we developed for calculations at this scale include a basis corrections for forces and strategies to identify “problematic” UCCSD­(T) structures, particularly those arising from spin symmetry breaking. We have further shown how UCCSD­(T) data sets of thousands of molecules together with transfer learning allows for the development of MLIPs for chemical reactions which show improved performance over MLIPs trained to DFT calculations and in a number of cases, improvements over DFT calculations themselves. The data set produced will be provided to the community upon publication of this work.

In the process of carrying out this work, a number of important considerations for the future of quantum chemistry and MLIP arose. First, it became clear that improving both the robustness and performance of software for QM calculations remains a vital pursuit for the advancement of MLIPs. This is necessary for the continued creation of highly accurate data sets at the CCSD­(T) level. The development of automated approaches to multireference calculations could provide a future direction for the automated construction of data sets for reactive processes as the limitations of UCCSD­(T) are known for structures with strong multireference character.^66–68^ Furthermore, the recent development of analytical force methods for local coupled cluster calculations paves the way for MLIPs to be able to recreate properties of larger molecules, including in condensed phase simulations.^69^

The expansion of the techniques developed in this work to the condensed phase is an open challenge that would greatly expand the applicability of the MLIP. This would require improvements in the sampling techniques used to construct the data sets, for example, so that small systems can be extracted and calculated at a higher level of theory,^70^ as well as further improvements in the high-level quantum chemistry methods applicable to condensed-phase systems.

Additionally, analysis of problematic structures in the data set revealed how automated sampling techniques produce reaction paths and transition states that are very unlikely to be produced by a chemist. There are benefits to this as the chemical space is not limited to known and predictable reactions. However, it does mean that the structures produced stretch quantum-chemistry methods to new, unseen limits. As a practical way forward, excluding structures that are unreliably described, as we have done, makes building organic molecule data sets simpler than carefully tailoring methods to an individual system.

We have taken the first steps to building a transferable reactive machine-learning potential that is capable of outperforming DFT and offers orders of magnitude better computational performance. By advancing toward UCCSD­(T)-level accuracy, MLIPs have the potential to revolutionize reactive simulations across diverse applications and offer unique insights into chemical reactivity on a large scale.

## Supplementary Material



## Notes

Additional details and validation of the basis set correction scheme, data filtering procedures, data set composition used to generate the UCCSD­(T)/QZ* training data, further benchmarking of the resulting MLIPs on bond dissociation curves, transition states, and minimum energy paths (PDF)
