# Supplementary material for: Reactive Chemistry at the Unrestricted Coupled Cluster Level: High-Throughput Calculations for Training Machine Learning Potentials
Source: J Chem Theory Comput. 2026 Jul 14;22(14):7286–96. doi: 10.1021/acs.jctc.6c00247 (PMC13422027; doi:10.1021/acs.jctc.6c00247)
Supplement: Supplementary file 1 [file ct6c00247_si_001.pdf]

Supporting Information:

Reactive Chemistry at Unrestricted Coupled Cluster Level: High-throughput Calculations for Training Machine Learning Potentials

Alice E. A. Allen,[*,†] Rui Li,[§] Sakib Matin,[†] Xing Zhang,[§] Benjamin Nebgen,[‡] Nicholas Lubbers,[‖] Justin S. Smith,[⊥] Richard Messerly,[‡] Sergei Tretiak,[‡] Garnet Kin-Lic Chan,[§] and Kipton Barros[†]

†*Center for Nonlinear Studies, Los Alamos National Laboratory, Los Alamos, New Mexico 87545, United States*

‡*Theoretical Division, Los Alamos National Laboratory, Los Alamos, New Mexico 87545, United States*

¶*Max Planck Institute for Polymer Research, Ackermannweg 10, 55128 Mainz, Germany,*

§*Division of Chemistry and Chemical Engineering, California Institute of Technology, Pasadena, 91125, CA, United States*

‖*Computer, Computational, and Statistical Sciences Division, Los Alamos National Laboratory, Los Alamos, New Mexico 87545, United States*

⊥*Nvidia Corporation, Santa Clara, CA 9505, United States*

#*National Center for Computational Sciences Division, Oak Ridge National Laboratory, Oak Ridge, TN 37830, United States*

@*Center for Integrated Nanotechnologies, Los Alamos National Laboratory, Los Alamos, New Mexico 87545, United States*

E-mail: allena@mpip-mainz.mpg.de

Contents

S1 Supplementary Methods **S3**

S1.1 UCCSD Data S3

S1.2 Basis Set Corrections S3

S1.3 Procedure for Identifying Possibly Inaccurate Structures S7

S1.3.1 Transition States, Reactants and Products S7

S1.3.2 The Dimer, NEB and Single-Ended String Structures S7

S1.4 Inconsistent Spin States for DZ/TZ/QZ S10

S2 Transition States, Products, Reactants **S11**

S2.1 DFT Datasets S11

S2.2 UCCSD Dataset S11

S2.3 UMP2 Dataset S13

S3 The NEB/Dimer/SEGS Datasets **S14**

S4 Dataset Sizes **S16**

S5 Fine-tuning MLIPs **S17**

S6 Machine Learning Potential Performance **S18**

S6.1 Bond Dissociation Curves S18

S6.2 UCCSD(T) Forces on Transition States Found with MLIPs S20

S6.3 Isomerization Reactions S21

S6.4 Malonaldehyde and Tropolone S23

References **S25**

S1 Supplementary Methods

S1.1 UCCSD Data

Whilst building the workflow for the AL process, UCCSD calculations were performed as an interim step between the DFT and UCCSD(T) calculations. The UCCSD/DZ calculations used the PySCF software with the cc-pVDZ basis set.[S1,S2] For completeness, the UCCSD data is included.

S1.2 Basis Set Corrections

We began by investigating the accuracy of basis set corrections for forces at the unrestricted CCSD(T) level. The computational cost of obtaining UCCSD(T)/TZ and UCCD(T)/QZ forces to benchmark against becomes extremely expensive for larger basis sets and memory requirements also become prohibitive. Given this, we begin by looking at the smallest molecules present in the dataset with just 6 atoms. Both reactants, products and transition states are included.

The fundamental assumption that we make in the basis set constructed is that the difference in forces between two basis sets (ie. between QZ and TZ) is correlated between MP2, UCCSD and UCCSD(T). We first demonstrate this is true. The difference between the TZ and DZ basis set forces for MP2(UCCSD) compared to the difference for UCCSD(T) is shown in Fig. S1 a) (Fig. S1 b)). The differences between the basis sets are highly correlated between the different levels of theory. A correction to UCCSD(T)/DZ using MP2/TZ and UCCSD/TZ forces is then shown in Fig. S1 c) and d). From this, we can see that using a UCCSD/TZ correction to the UCCSD(T)/DZ basis is approximately 50% more accurate than an MP2 correction. Additionally, even for the larger forces present the UCCSD correction remains accurate, whereas for the MP2 correction larger deviation appear at larger forces. We can also see from Fig. S1 a) and b) that the basis set correction from DZ to TZ is quite large, with a maximum value of 2.31 and 2.55 for MP2 and UCCSD respectively -

this is also shown for MP2 in Fig. S1 e). This shows the importance of basis set corrections.

Even for six atom molecules, analytical UCCSD(T)/QZ forces cannot be readily computed with PySCF. However, the forces can be compared for MP2/QZ and MP2/TZ as shown in Fig. S1 d). This figure demonstrates that the MP2/QZ and MP2/TZ forces are much more highly correlated than MP2/TZ and MP2/DZ forces.

To correct to the QZ basis set limit, we propose a new method and use the MP2/QZ forces in addition to the UCCSD/TZ correction. The forces are:

$F_{UCCSD(T)/QZ*} = F_{UCCSD(T)/DZ} + (F_{UCCSD/TZ} - F_{UCCSD/DZ}) + (F_{UMP2/QZ} - F_{UMP2/TZ})$.

The accuracy of this correction was tested on a set of molecules containing configurations of CO_2, H_2O, C_2H_2 and H_2CO generated using 300K MD. The results are shown in Fig. S2 with the proposed correction of UCCSD and MP2 offering a higher accuracy than correcting just to the TZ level with UCSSD or using MP2 alone to correct the force to the QZ basis set - see Fig. S2. The corrected basis set is described as UCCSD(T)/QZ* throughout this work. The UCCSD calculations are corrected from the DZ basis set using MP2 alone and is described as UCCSD/QZ*.

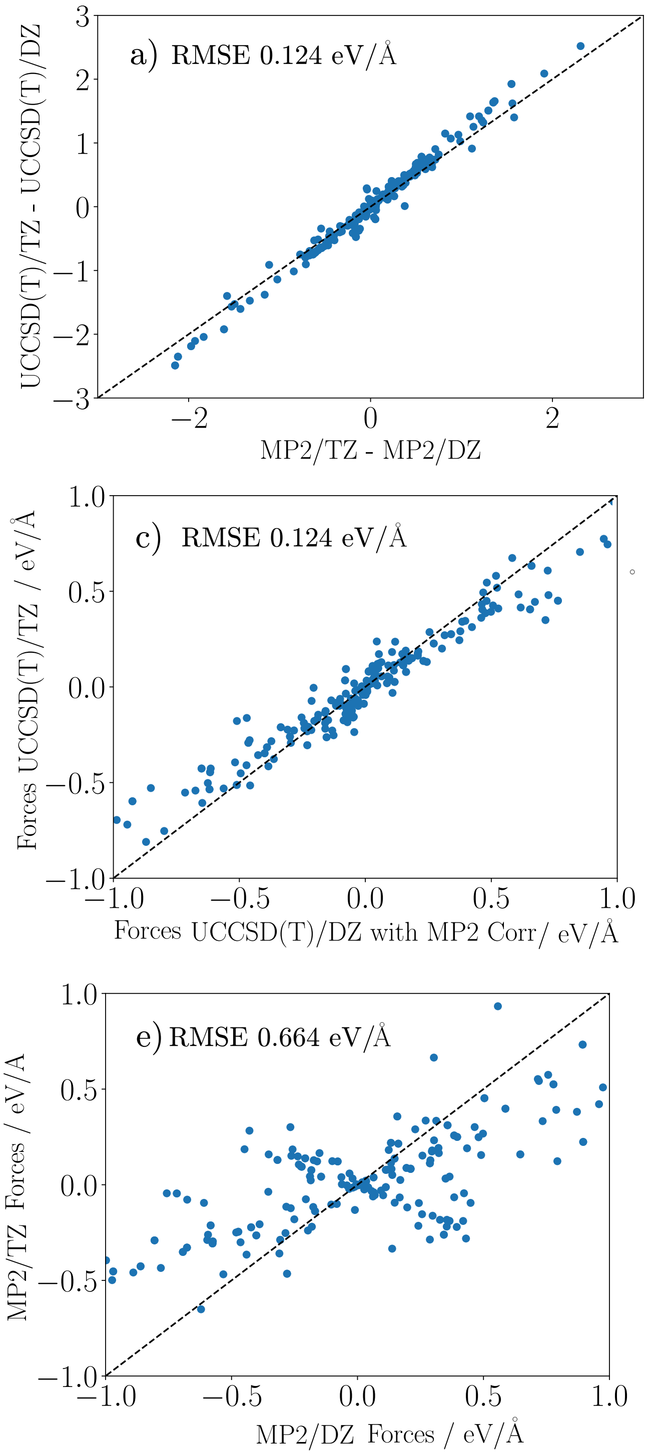

Figure S1: The difference between UCCSD(T) forces with a TZ and DZ basis set compared to the difference between the TZ and DZ forces with a) MP2 and b) UCCSD respectively. The accuracy of basis set corrections to UCCSD(T)/DZ with c) MP2 and d) UCCSD compared to the UCCSD(T)/TZ forces is then shown. A comparison of the MP2 TZ forces with DZ is shown in part d) and MP2 QZ forces with TZ is show in part e). The dataset used contains both reactant, products and transition states.

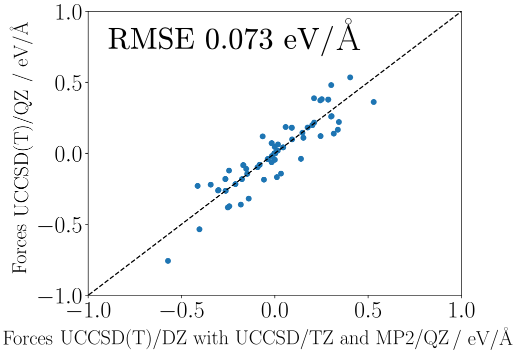

Figure S2: The difference between UCCSD(T)/QZ forces and UCCSD(T)/QZ* forces. The UCCSD(T)/QZ* forces contain a basis set correction with a UCCSD/TZ and MP2/QZ calculation performed.

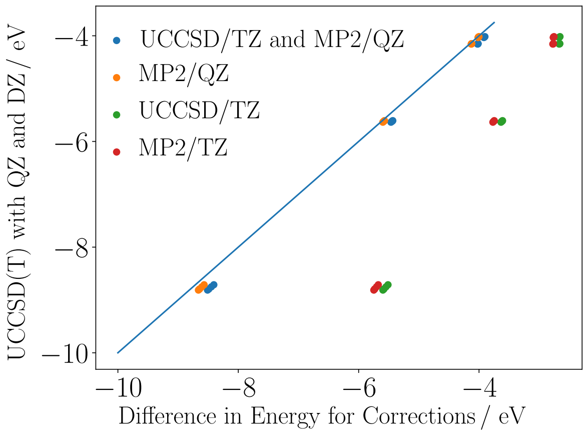

Figure S3: The difference in energy between a QZ and DZ basis set at the UCCSD(T) level is compared to the difference in energy with various approximations. The UMP2(UCCSD)/TZ data points correspond to the difference between UMP2(UCCSD)/TZ and UMP2(UCCSD)/DZ with the UCCSD(T) data. The UMP2/QZ data points correspond to the difference between UMP2/QZ and UMP2/DZ with the UCCSD(T) data. The UCCSD/TZ and UMP2/QZ data points correspond to the difference between (UCCSD/TZ - UCCSD/DZ + UMP2/QZ - UMP2/TZ) with the UCCSD(T) data.

S1.3 Procedure for Identifying Possibly Inaccurate Structures

S1.3.1 Transition States, Reactants and Products

As discussed, cases were found where extremely large forces were present for the calculation to be performed. To overcome this, a procedure was developed to identify structures whose forces may be particularly large. Alongside the unrestricted calculations, if the spin state was non-zero then a restricted calculation was also performed. If the restricted calculation was lower energy than the unrestricted calculation, or the difference between the two was less than 0.1eV, the structure was identified as possibly being near an intersection point of spin states. For the transition states, reactants and products, if the difference in forces was greater than 1eV/Å, these points were then removed. Additionally, for all structures if the force basis set correction was greater that 1eV/Å for the MP2 component then the structure was also not included. For the dataset containing products, reactants and transition states, this resulted in 114 calculations being removed from the main dataset due to this procedure. A comparison of the filtered and unfiltered forces for this dataset are shown in Fig. S10. There are a number of possible causes for the behaviour seen. For example, spin-contaminated solution from HF may distort the PES obtained from CCSD(T), and hence cause the atomic gradient to become erroneous.

S1.3.2 The Dimer, NEB and Single-Ended String Structures

The structures sampled along the reaction path are further from the intersection point and the coupled cluster calculations are less problematic. Therefore, for these cases, the structures were removed if the basis set correction for the forces contained a component greater than 5 eV/Å for the UCCSD component and greater than 1 eV/Å for the UMP2 component.

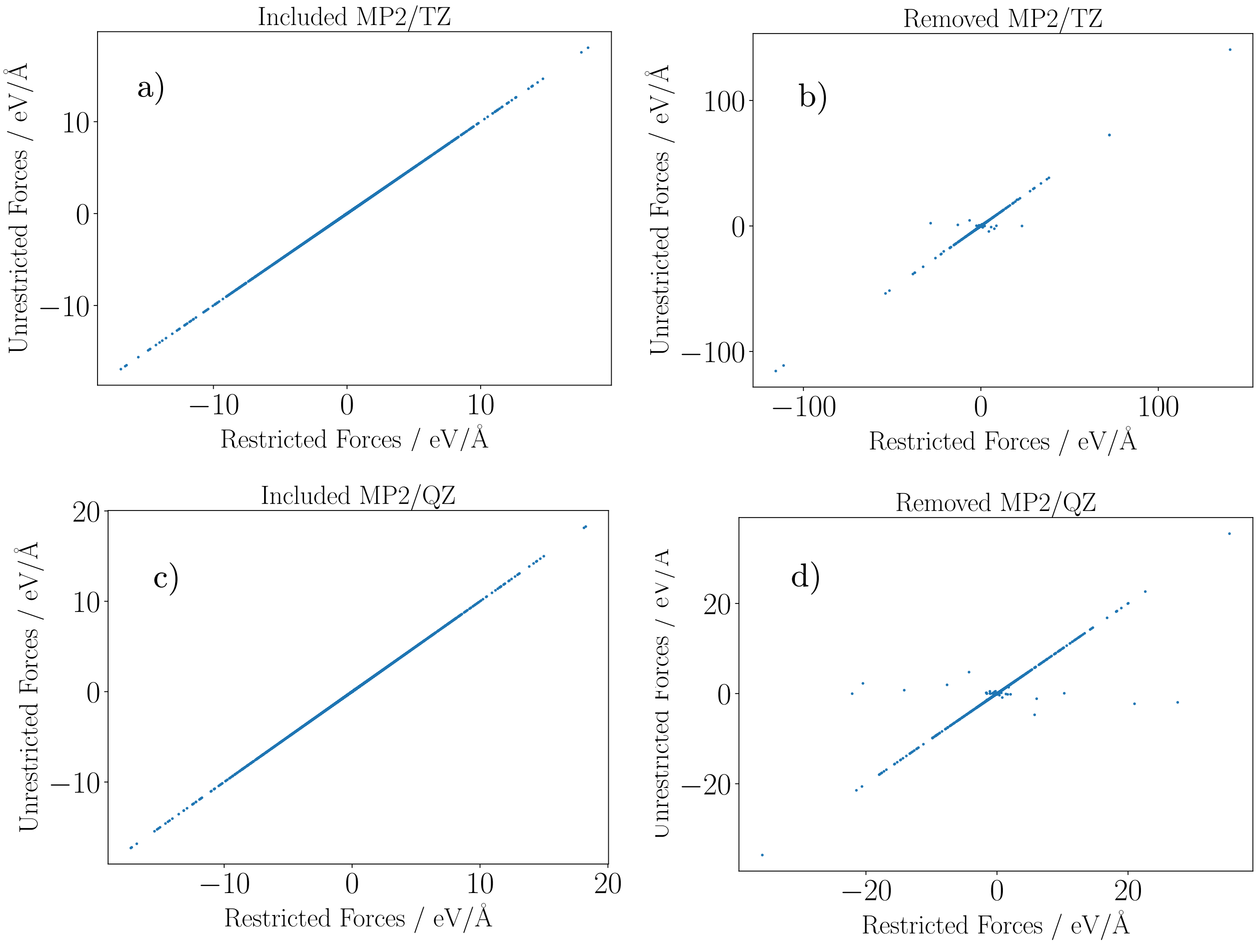

A comparison of the unrestricted and restricted forces for the included and removed structures for MP2 level of theory.

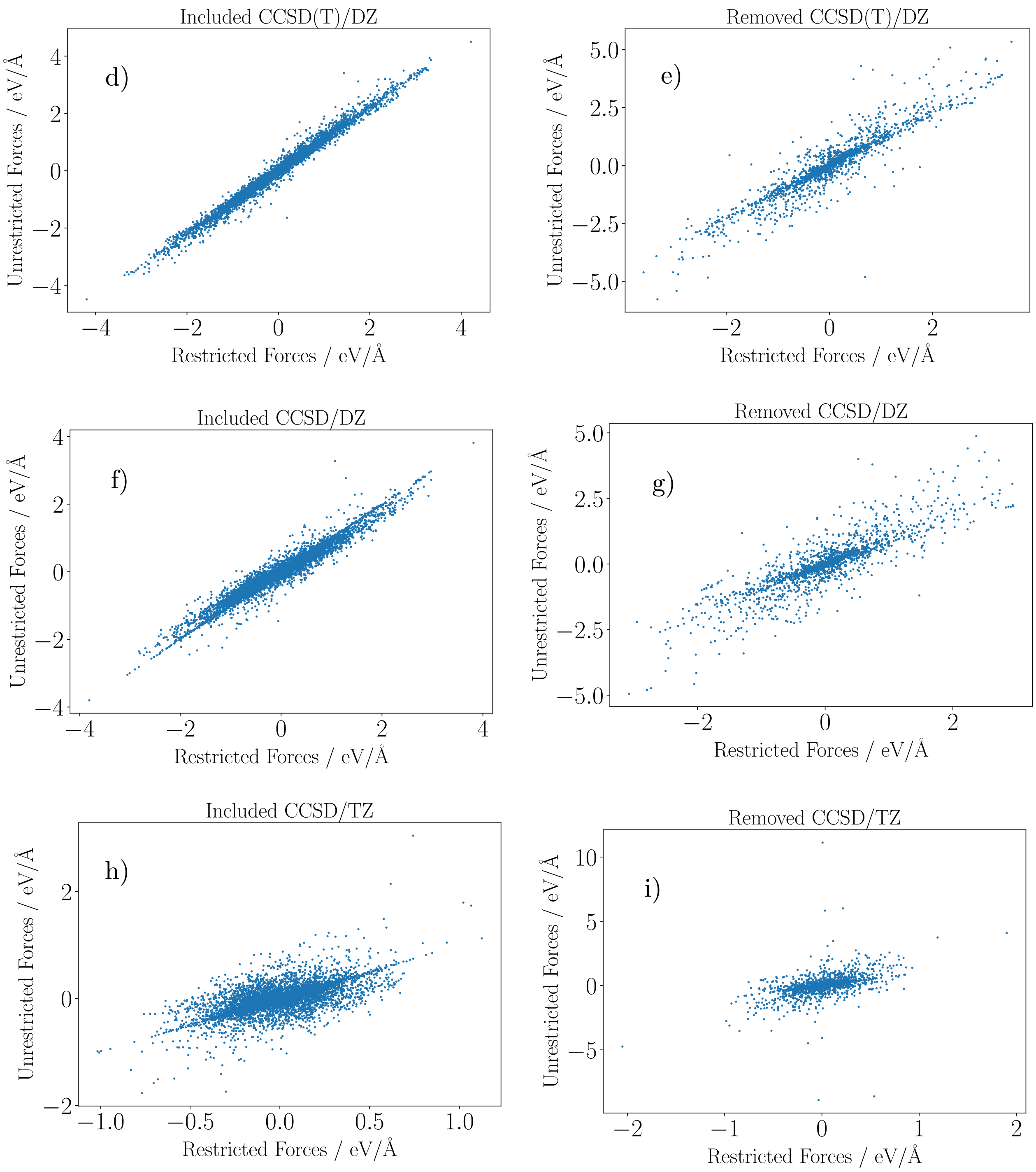

Figure S4: A comparison of the unrestricted and restricted forces for the included and removed structures for CCSD and CCSD(T) level of theory.

S1.4 Inconsistent Spin States for DZ/TZ/QZ

We also identified that inconsistent spin states for the DZ/TZ/QZ basis states found with the automated searching method resulted in incorrect calculations. To analyze the disparities present, we show a comparison of the $\langle S^2 \rangle$ values for the different basis sets in Fig. S10. Structures with $|\langle S^2 \rangle_{QZ} - \langle S^2 \rangle_{TZ}| > 0.1$ or $|\langle S^2 \rangle_{QZ} - \langle S^2 \rangle_{DZ}| > 0.1$ were removed.

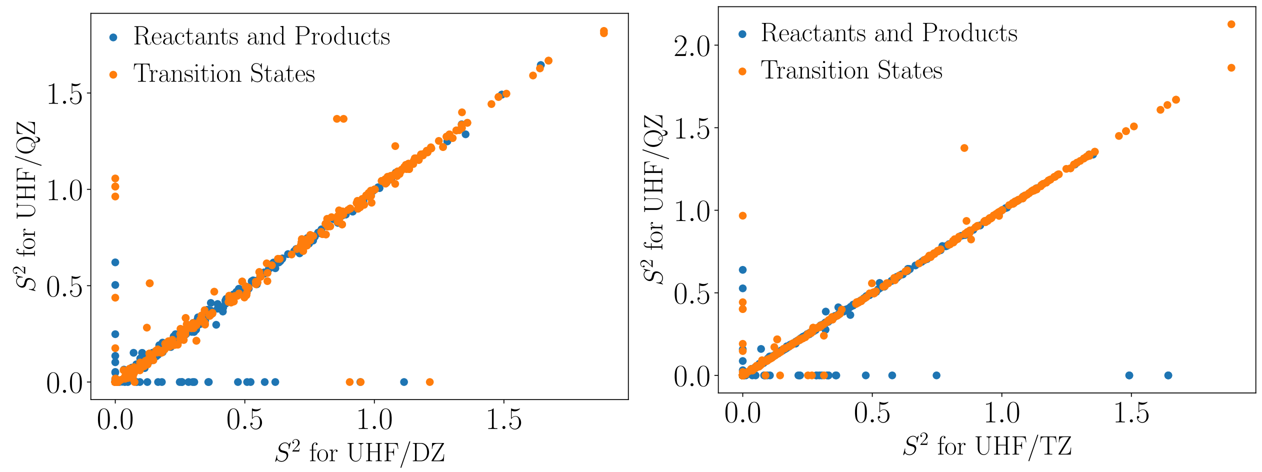

Figure S5: A comparison between the UHF/QZ spin states with the UHF/TZ and UHF/DZ spin states for the reactants, products and transition states.

S2 Transition States, Products, Reactants

Further analysis of the transition states, products and reactants is given in this section for various levels of theory.

S2.1 DFT Datasets

For the DFT datasets, additional analysis of the error distribution for the forces was performed.

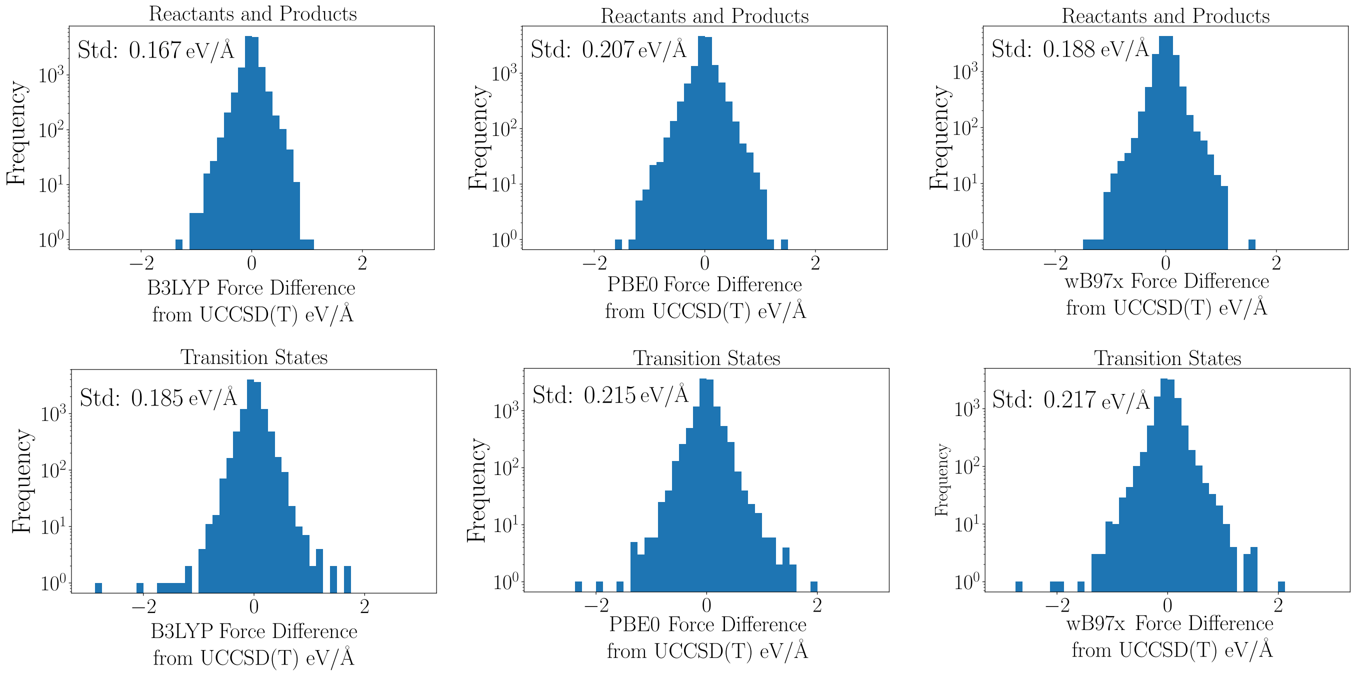

Figure S6: The distributions of force differences between the unrestricted CCSD(T)/QZ* level of theory and various levels of DFT. The mean value of all the distributions is 0.00.

S2.2 UCCSD Dataset

An analysis of the forces for the UCCSD level of theory is shown. Large differences in the TZ and DZ forces are seen in Fig. S7. The advantages of using UCCSD(T) level of theory over UCCSD are seen in Fig. S10.

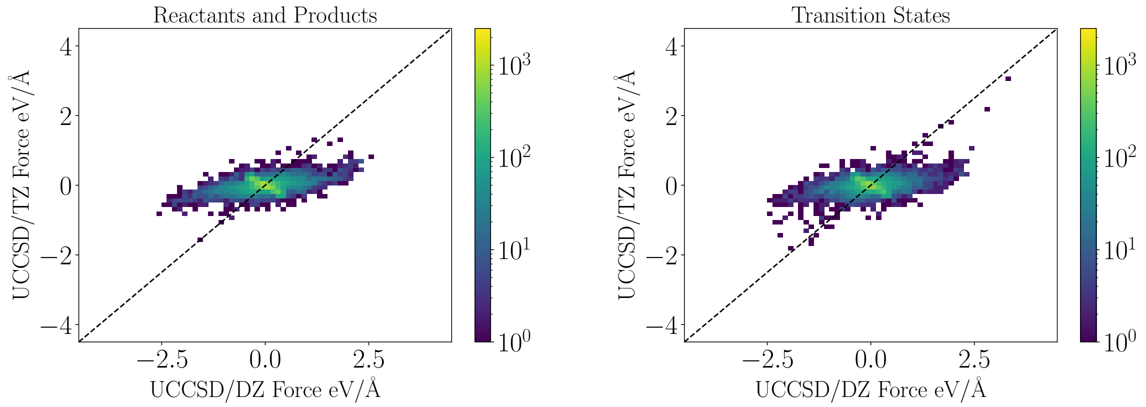

Figure S7: A comparison between the unrestricted CCSD level of theory for DZ and TZ basis sets.

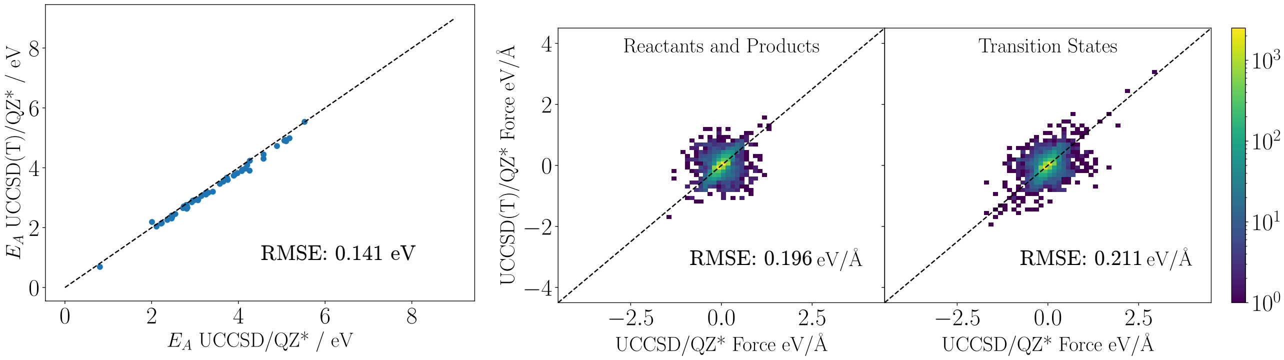

Figure S8: A comparison of the unrestricted CCSD(T)/QZ* level of theory and unrestricted CCSD/QZ* for the E_A and forces.

S2.3 UMP2 Dataset

A comparison of the forces for the UMP2 dataset with basis set is given in Fig. S13. The difference between the UMP2/QZ E_A and forces and UCCSD(T)/QZ* E_A and forces is shown in Fig. S10.

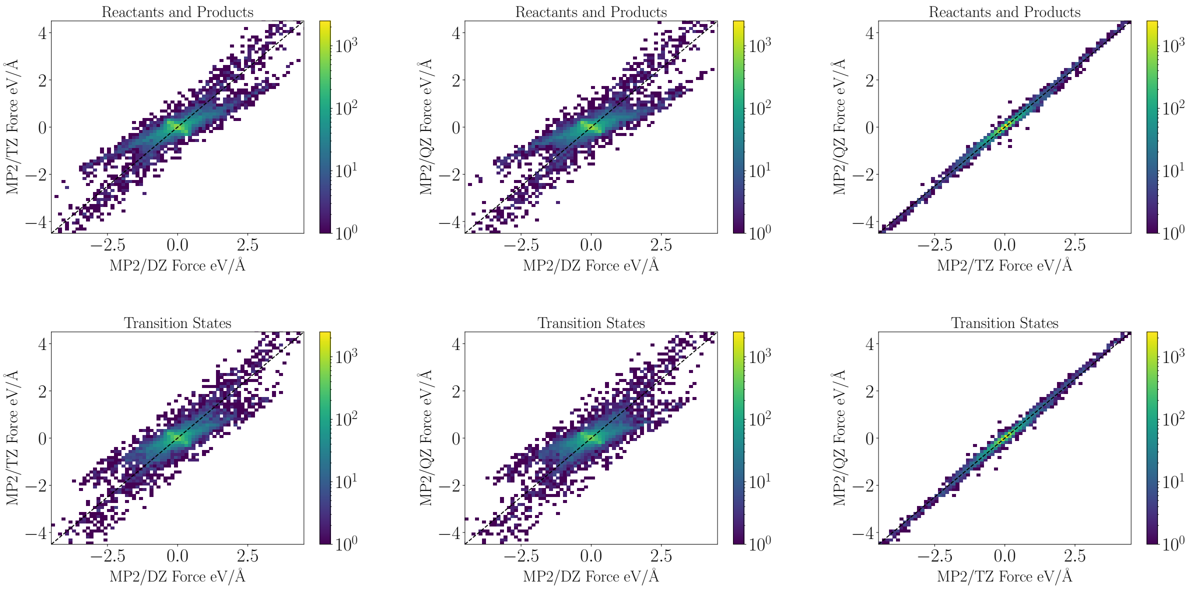

Figure S9: A comparison between the unrestricted MP2 level of theory for DZ, TZ and QZ basis sets.

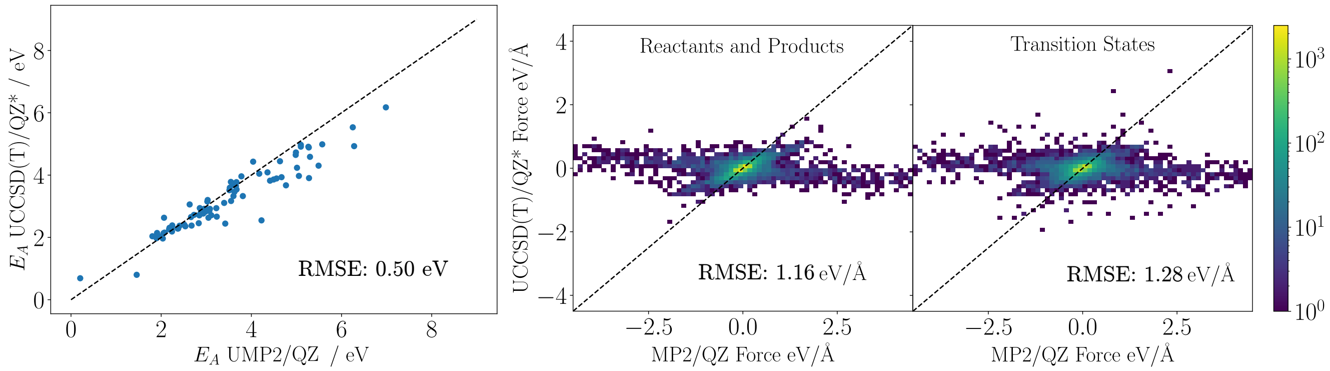

Figure S10: A comparison between the unrestricted CCSD(T)/QZ* level of theory and unrestricted MP2/QZ for the E_A and forces.

S3 The NEB/Dimer/SEGS Datasets

Additional analysis of the NEB/Dimer/SEGS dataset for the DFT functionals and UCCSD(T)/QZ* is given in Fig. S12 and Fig. S11.

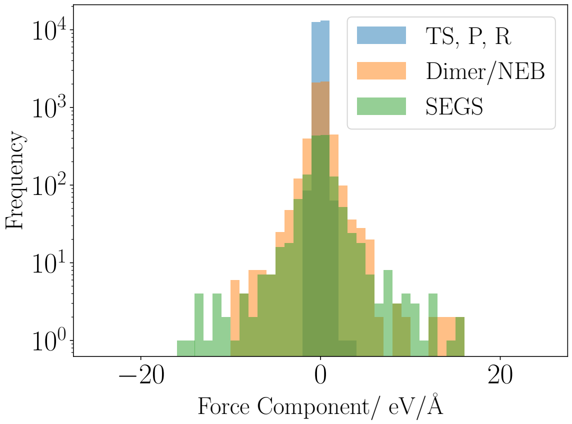

Figure S11: The distribution of UCCSD(T)/QZ* force components for the transition states, products and reactants (TS, P, R), the dimer and NEB structures and SEGS. A wider distribution of forces is present for the dimer, NEB and SEGS structures.

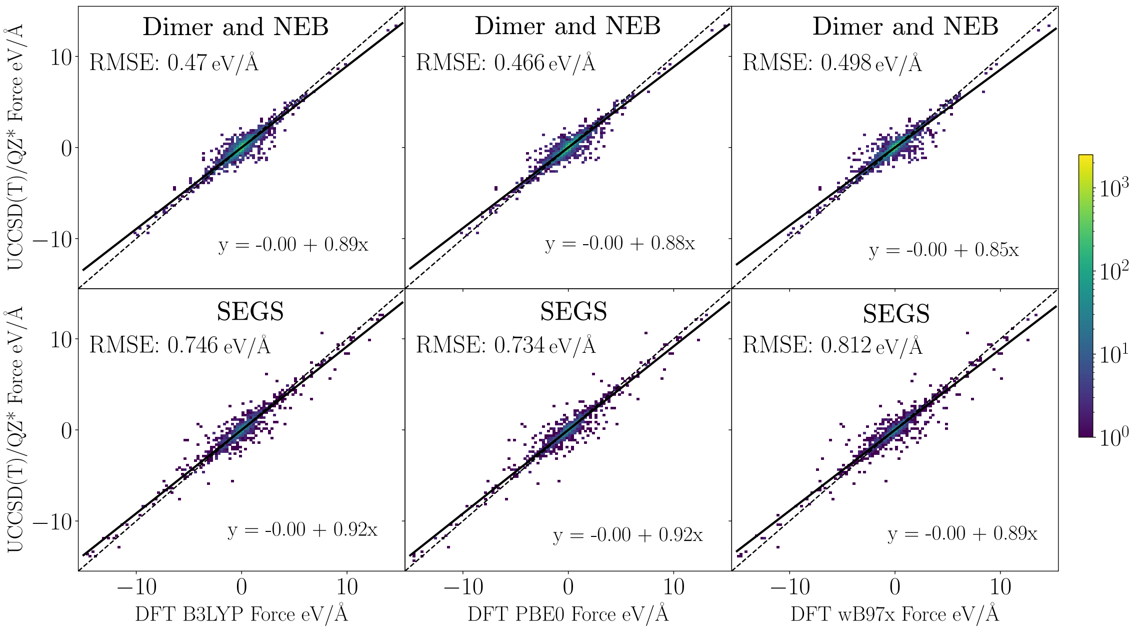

Figure S12: A comparison in forces between the DFT functionals calculated in the singlet state and UCCSD(T)/QZ* for NEB, the dimer method and the SEGS.

S4 Dataset Sizes

A summary of the size of each dataset created is presented in this section. First, an analysis by the level of theory used is provided, followed by a categorization based on the sampling technique for the UCCSD(T)/QZ* data.

Table S1: The number of calculations performed and provided at DFT/TZ, UCCSD/DZ and UCCSD(T)/QZ* levels of theory.

**QM Method**	**Number of Structures**
ωB97X-D3/def2-TZVP	270720
UCCSD/DZ	30154
*UCCSD(T)/QZ**	3119

Furthermore, a breakdown of the sampling techniques for the UCCSD(T)/QZ* is given. It should be noted that the number of structures alone can give a misleading indication of the importance of the sampling technique when fitting MLIPs. The bond dissociation structures are much smaller than the other sampling techniques and therefore provide a smaller fraction of the forces than otherwise indicated.

Table S2: The number of calculations performed for each sampling method for the UCCSD(T)/QZ* dataset.

**Sampling Method**	**Number of Structures**
Transition States, Reactants, Products	950
NEB/Dimer	167
SEGS	49
Bond Dissociation	1953

S5 Fine-tuning MLIPs

Fine-tuning of the HIP-HOP-NN model did not freeze any parameters and maintained the same training and architecture hyperparameter used for the HIP-HOP-NN DFT model.

The fine-tuning of the MACE-MP medium potential to the UCCSD(T) dataset used the run_train.py python command with the following settings:

foundation_model=medium

multiheads_finetuning=False

train_file="All_UCCSDT_train.xyz"

valid_file="All_UCCSDT_valid.xyz"

test_file="All_UCCSDT_test.xyz"

E0s="1:-13.60421,6:-1028.91941,7:-1484.46528,8:-2041.48145"

lr=0.001

batch_size=10

max_num_epochs=150

device=cuda

default_dtype="float64"

energy_key='energy'

forces_key='forces'

clip_grad=10000

loss="weighted"

forces_weight=1000

energy_weight=10

swa

start_swa=100

swa_lr=0.000001

S6 Machine Learning Potential Performance

Additional results for the machine learning potential trained to the UCCSD(T)/QZ* data is given.

S6.1 Bond Dissociation Curves

The bond dissociation curves for hydrogen bond dissociation for QM9 molecules are shown in Fig. S13.

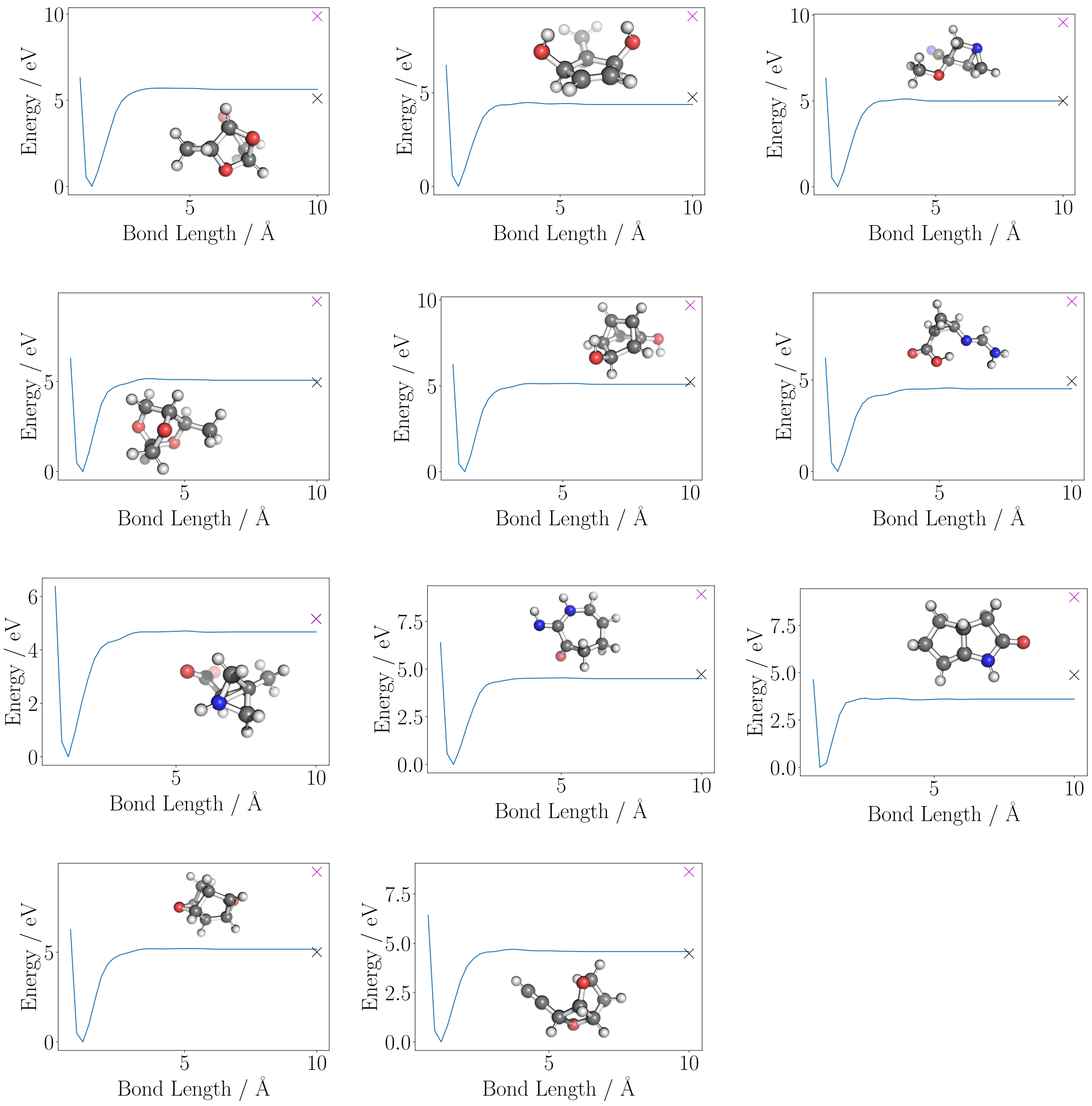

Figure S13: The hydrogen bond dissociation energies given by the MLIP for randomly chosen molecules from the QM9. The black cross shows the UCCSD(T) bond dissociation energy relative to the equilibrium value. The magenta cross shows the DFT singlet bond dissociation energy. All of the molecules chosen are larger than the largest molecule used in the training dataset and rigid scans are performed.

S6.2 UCCSD(T) Forces on Transition States Found with MLIPs

The UCCSD(T)/QZ* forces for the TS found with the MLIP trained to the UCCSD(T)/QZ* dataset are shown in Fig. S15. Whilst DFT recreates UCCSD(T) transition states forces better for this subset, it is orders of magnitude slower. A comparison of the DFT and MLIP TS found are shown in Fig. S14.

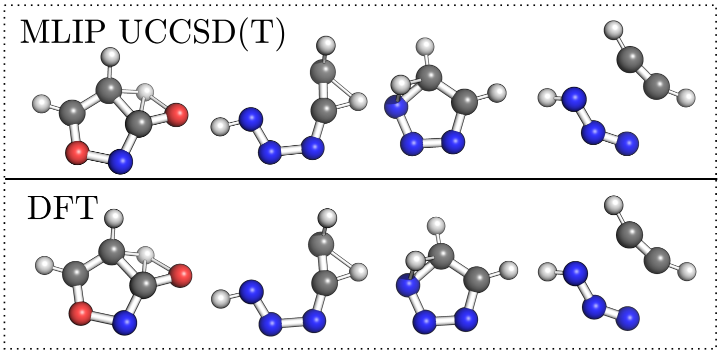

Figure S14: Transition state structures found with the dimer method using DFT and the MLIP trained to UCCSD(T) data are shown.

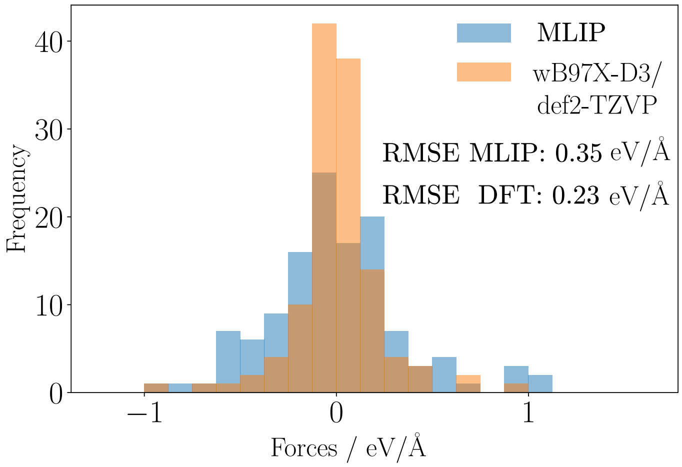

Figure S15: The distribution of forces for transition states found using the dimer method using different potentials.

S6.3 Isomerization Reactions

The minimum energy path for isomerization reactions are shown in Fig. S16 and in Fig. S17.

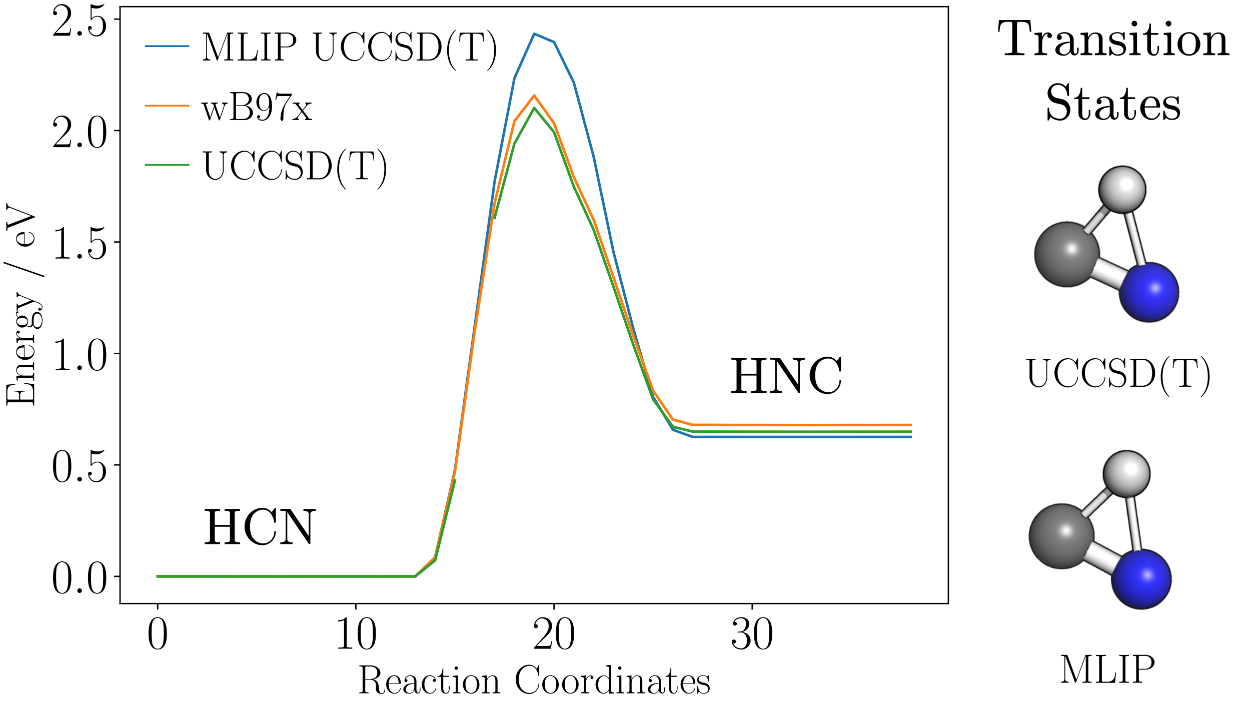

Figure S16: The minimum energy path found with the nudged elastic band (NEB) technique using the HIP-HOP MLIP for HCN to HNC. The transition states for the MLIP and for UCCSD(T) found with the dimer method are also shown.

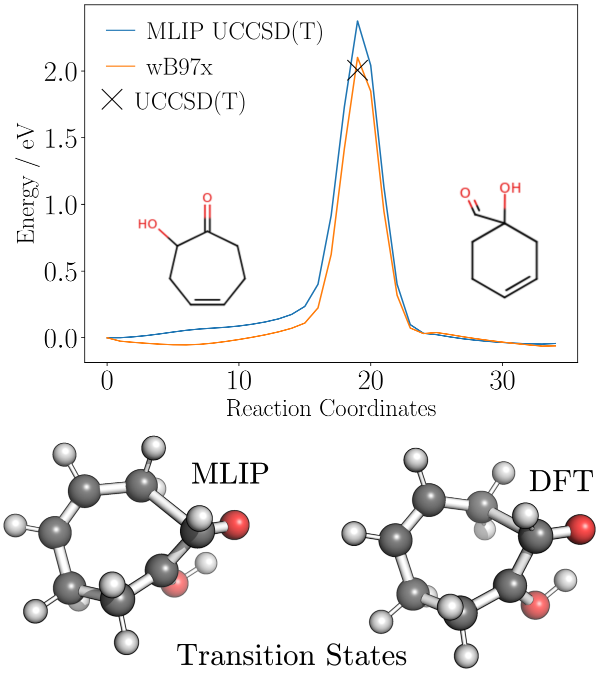

Figure S17: The minimum energy path found with the NEB technique using the HIP-HOP MLIP for the cyclic isomerization reaction from Ref. 53. The black cross shows the energy difference between the product and transition state for UCCSD(T). The transition states with the MLIP trained at UCCSD(T) is compared to the DFT structure from Ref. 53.

S6.4 Malonaldehyde and Tropolone

The MEP path for the proton transfer in malonaldehyde was found using NEB and is shown in Fig. S18. The MEP shows a smooth path and expected transition of the proton. However, the HIP-HOP-NN underestimates the energies in comparison to the UCCSD(T)/QZ* calculations.

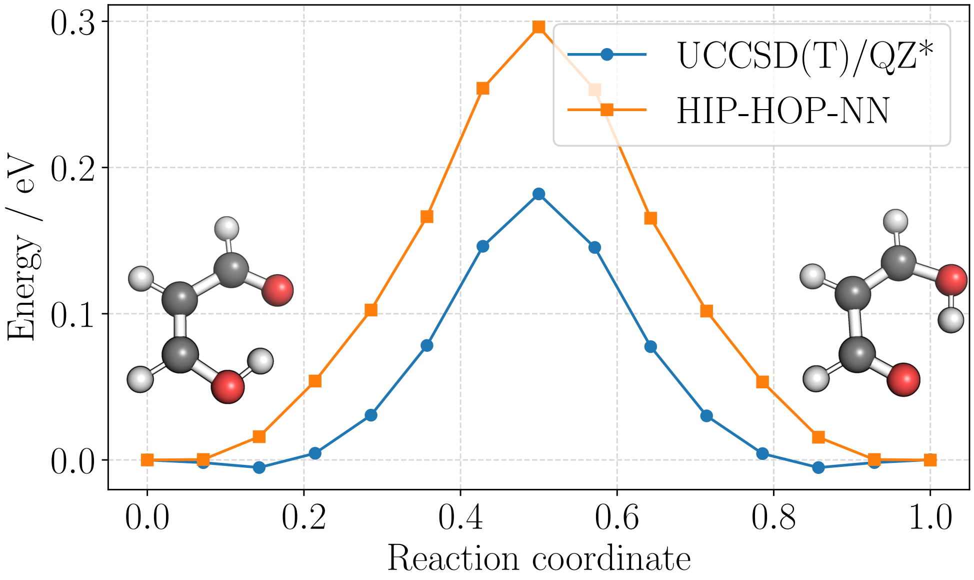

Figure S18: The minimum energy path found with the NEB technique using HIP-HOP-NN trained to UCCSD(T) data for the transfer of a proton in malonaldehyde. The UCCSD(T)/QZ* energies for the HIP-HOP-NN MEP are shown.

Additionally, molecular dynamics simulations were performed using a Langevin thermostat as implemented in ASE, with a timestep of 0.1 fs, a temperature of 500 K, and a friction coefficient of 0.01 fs^{-1}. A 100ps simulation was performed. The molecular dynamics simulation for malonaldehyde indicated that hydrogen atom transfer occurred at 500 K whilst the rest of the molecule remained stable.

Similarly, for tropolone the MEP path for proton transfer was found and is shown in Fig. S19. UCCSD(T)/DZ calculations are compared to the MLIP rather than UCCSD(T)/QZ* as the computational costs of UCCSD(T)/QZ* was too high. Reactive molecular dynamics for tropolone showed a stable tropolone molecule with hydrogen atom transfer occurring at 500K.

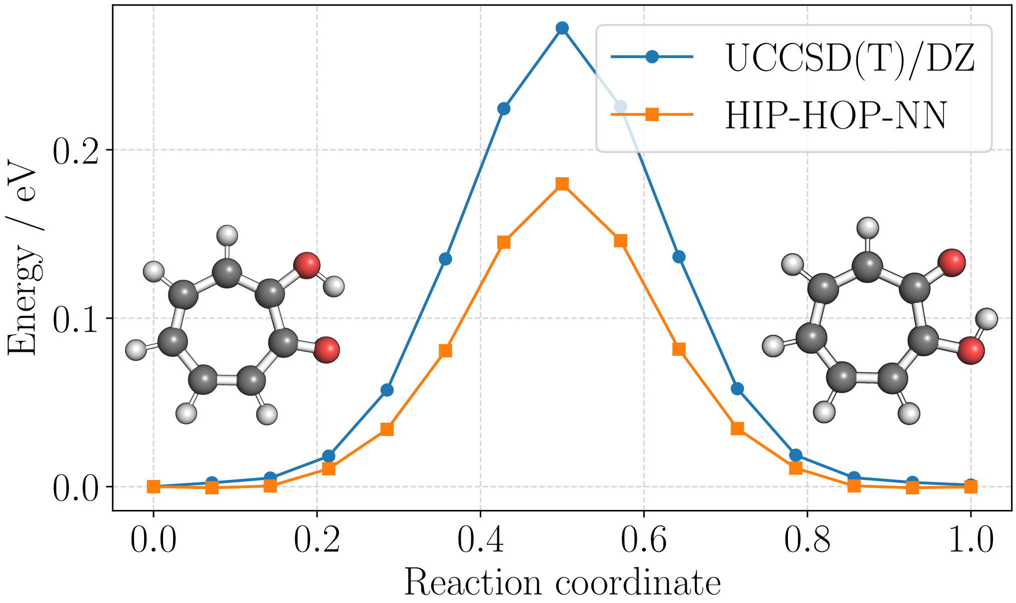

Figure S19: The minimum energy path found with the NEB technique using HIP-HOP-NN trained to UCCSD(T) data for the transfer of a proton in tropolone. The UCCSD(T)/DZ energies for the HIP-HOP-NN MEP are shown.

References

(S1) Sun, Q.; Zhang, X.; Banerjee, S.; Bao, P.; Barbry, M.; Blunt, N. S.; Bogdanov, N. A.; Booth, G. H.; Chen, J.; Cui, Z.-H.; Eriksen, J. J.; Gao, Y.; Guo, S.; Hermann, J.; Hermes, M. R.; Koh, K.; Koval, P.; Lehtola, S.; Li, Z.; Liu, J.; Mardirossian, N.; McClain, J. D.; Motta, M.; Mussard, B.; Pham, H. Q.; Pulkin, A.; Purwanto, W.; Robinson, P. J.; Ronca, E.; Sayfutyarova, E. R.; Scheurer, M.; Schurkus, H. F.; Smith, J. E. T.; Sun, C.; Sun, S.-N.; Upadhyay, S.; Wagner, L. K.; Wang, X.; White, A.; Whitfield, J. D.; Williamson, M. J.; Wouters, S.; Yang, J.; Yu, J. M.; Zhu, T.; Berkelbach, T. C.; Sharma, S.; Sokolov, A. Y.; Chan, G. K.-L. Recent developments in the PySCF program package. *J. Chem. Phys.* **2020**, *153*, 024109.

(S2) Sun, Q.; Berkelbach, T. C.; Blunt, N. S.; Booth, G. H.; Guo, S.; Li, Z.; Liu, J.; McClain, J. D.; Sayfutyarova, E. R.; Sharma, S.; Wouters, S.; Chan, G. K.-L. PySCF: the Python-based simulations of chemistry framework. *WIREs Comput. Mol. Sci.* **2018**, *8*, e1340.
